# Evolutionary diversification of C_2_ photosynthesis in the grass genus *Homolepis* (Arthropogoninae)

**DOI:** 10.1093/aob/mcae214

**Published:** 2024-12-17

**Authors:** Joyce Pereira Alvarenga, Matt Stata, Rowan F Sage, Ria Patel, Ane Marcela das Chagas Mendonca, Felipe Della Torre, Hongbing Liu, Shifeng Cheng, Samantha Weake, Emile J Watanabe, Pedro Lage Viana, Iago Augusto de Castro Arruda, Martha Ludwig, João Paulo Rodrigues Alves Delfino Barbosa, Tammy L Sage

**Affiliations:** Laboratory of Ecophysiology, Plant Physiology Sector, Department of Biology, Federal University of Lavras, Lavras, Minas Gerais, 37200-900, Brazil; Department of Ecology and Evolutionary Biology, University of Toronto, Ontario, M5S 3B2, Canada; Department of Ecology and Evolutionary Biology, University of Toronto, Ontario, M5S 3B2, Canada; Department of Ecology and Evolutionary Biology, University of Toronto, Ontario, M5S 3B2, Canada; Department of Ecology and Evolutionary Biology, University of Toronto, Ontario, M5S 3B2, Canada; Laboratory of Ecophysiology, Plant Physiology Sector, Department of Biology, Federal University of Lavras, Lavras, Minas Gerais, 37200-900, Brazil; Department of Ecology and Evolutionary Biology, University of Toronto, Ontario, M5S 3B2, Canada; Department of Ecology and Evolutionary Biology, University of Toronto, Ontario, M5S 3B2, Canada; Laboratory of Plant Physiology, Department of Botany, Institute of Science Biology, Federal University of Minas Gerais, Belo Horizonte, Minas Gerais, 31270-901, Brazil; Shenzhen Branch, Guangdong Laboratory for Lingnan Modern Agriculture, Genome Analysis Laboratory of the Ministry of Agriculture, Agricultural Genomics Institute at Shenzhen, Chinese Academy of Agricultural Sciences, Shenzhen, 518120, China; Shenzhen Branch, Guangdong Laboratory for Lingnan Modern Agriculture, Genome Analysis Laboratory of the Ministry of Agriculture, Agricultural Genomics Institute at Shenzhen, Chinese Academy of Agricultural Sciences, Shenzhen, 518120, China; Department of Ecology and Evolutionary Biology, University of Toronto, Ontario, M5S 3B2, Canada; Department of Ecology and Evolutionary Biology, University of Toronto, Ontario, M5S 3B2, Canada; Instituto Nacional da Mata Atlantica, Santa Teresa, Espirito Santo, 29650-000, Brazil; Laboratory of Ecophysiology, Plant Physiology Sector, Department of Biology, Federal University of Lavras, Lavras, Minas Gerais, 37200-900, Brazil; School of Molecular Sciences, University of Western Australia, Crawley, WA 6009, Australia; Laboratory of Ecophysiology, Plant Physiology Sector, Department of Biology, Federal University of Lavras, Lavras, Minas Gerais, 37200-900, Brazil; Department of Ecology and Evolutionary Biology, University of Toronto, Ontario, M5S 3B2, Canada

**Keywords:** Carbon concentrating mechanism, C_3_–C_4_ intermediate, C_2_ photosynthesis, C_4_ photosynthesis, glycine decarboxylase, leaf anatomy, photosynthetic evolution

## Abstract

**Background and Aims:**

To better understand C_4_ evolution in monocots, we characterized C_3_–C_4_ intermediate phenotypes in the grass genus *Homolepis* (subtribe Arthropogoninae).

**Methods:**

Carbon isotope ratio (δ^13^C), leaf gas exchange, mesophyll (M) and bundle sheath (BS) tissue characteristics, organelle size and numbers in M and BS tissue, and tissue distribution of the P-subunit of glycine decarboxylase (GLDP) were determined for five *Homolepis* species and the C_4_ grass *Mesosetum loliiforme* from a phylogenetic sister clade. We generated a transcriptome-based phylogeny for *Homolepis* and *Mesosetum* species to interpret physiological and anatomical patterns in an evolutionary context, and to test for hybridization.

**Key Results:**

*Homolepis* contains two C_3_ species (*H*. *glutinosa*, *H*. *villaricensis*), one species with a weaker form of C_2_ termed sub-C_2_ (*H. isocalycia*), and two C_2_ species (*H. longispicula*, *H. aturensis*). *Homolepis longispicula* and *H. aturensis* express over 85 % of leaf glycine in centripetal mitochondria within the BS, and have increased fractions of leaf chloroplasts, mitochondria and peroxisomes within the BS relative to *H. glutinosa*. Analysis of leaf gas exchange, cell ultrastructure and transcript expression show *M. loliiforme* is a C_4_ plant of the NADP-malic enzyme subtype. *Homolepis* comprises two sister clades, one containing *H. glutinosa* and *H. villaricensis* and the second *H. longispicula* and *H. aturensis. Homolepis isocalycia* is of hybrid origin, its parents being *H. aturensis* and a common ancestor of the C_3_  *Homolepis* clade and *H. longispicula*.

**Conclusions:**

Photosynthetic activation of BS tissue in the sub-C_2_ and C_2_ species of *Homolepis* is similar to patterns observed in C_3_–C_4_ intermediate eudicots, indicating common evolutionary pathways from C_3_ to C_4_ photosynthesis in these disparate clades. Hybridization can diversify the C_3_–C_4_ intermediate character state and should be considered in reconstructing putative ancestral states using phylogenetic analyses.

## INTRODUCTION

**Fig. 1.**
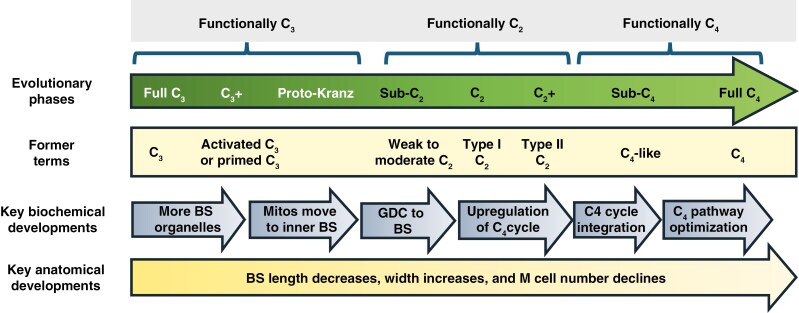
Schematic outlining key phases of C_4_ evolution based upon patterns identified in eudicot species, principally within the Asteraceae genus *Flaveria*. The diagram is modified from Stata *et al.* (2019) and the nomenclatural update in Adachi *et al.* (2023). See Sage *et al.* (2012, 2014), Heckmann *et al.* (2013), Mallman *et al.* (2014), Stata *et al*. (2019) and Leung *et al*. (2024) for further discussion and rationale for the current scheme. Mitos, mitochondria. The scheme presents C_4_ evolution as a series of phases beginning with the transition from fully C_3_ photosynthesis, where BS cells are typically small with low organelle numbers, to an augmented C_3_ state (C_3_+), where there is an enhancement of organelle numbers in the BS. This enhancement increases the capacity of C_3_ photosynthesis in BS cells and is often associated with enlarged BS cells. Following BS augmentation, mitochondria and some chloroplasts localize along the inner, centripetal wall of the BS cells, adjacent to the vascular tissue, creating a distinct character state that has been termed proto-Kranz (Muhaidat *et al.*, 2011; Sage *et al*, 2013). In many C_3_ grasses, most mitochondria are already in a centripetal position in the BS (Hatakeyama and Ueno, 2016), such that the key development during the evolution of the proto-Kranz trait would be repositioning nearly all BS mitochondria centripetally. Often, the mitochondrial shift is accompanied by an increased radial appearance of the BS in cross-section, giving the appearance of an incipient version of Kranz anatomy. The proto-Kranz and C_3_ + phenotypes are C_3_ in function because there is minimal shift of GDC expression from M to BS cells. Once GDC expression begins to shift to the BS cells, a photorespiratory glycine shuttle can operate, marking the inception of C_2_ function and the beginning of the sub-C_2_ character state. As proportionally more GDC is expressed in BS cells, the C_2_ pathway strengthens, until GDC is minimally expressed in the M tissue, giving a full C_2_ phenotype. Initially, full C_2_ photosynthesis may be accompanied by little activity of C_4_ cycle components, in what was formerly termed Type I C_3_–C_4_ intermediacy (Edwards and Ku, 1987). Elements of the C_4_ cycle are upregulated in full C_2_ species, probably to enhance recycling of photorespiratory N back to the M tissues following its release by glycine decarboxylation in the BS mitochondria (Mallmann *et al*., 2014; Walsh *et al.*, 2023). In the C_2_+ phenotype, a moderate C_4_ cycle functions but is poorly integrated with C_3_ metabolism in the BS tissue (Monson *et al*., 1986). Strong integration of the C_3_ and C_4_ cycles is considered a key criterion for C_4_ photosynthesis because it allows for rapid refixation of most CO_2_ transported from M to BS cells (Edwards *et al.*, 2001). The initial phase of a functional C_4_ pathway is defined by the integrated co-function of the C_3_ and C_4_ cycles; however, it is also associated with residual expression of C_3_ metabolism in M tissues, and a lack of optimized enzyme kinetics and regulatory integration for efficient C_4_ function (Monson and Rawsthorne, 2000). We call this functional but incomplete C_4_ state the sub-C_4_ phase. The final phase of C_4_ evolution is characterized by the optimization of regulatory integration and compartmentalization between M and BS tissues, and improved coordination of the altered photosynthetic performance with the carbon metabolism of the entire plant. *Nomenclature rationale*: the nomenclature proposed here clarifies the description of evolutionary phases between C_3_ and C_4_ photosynthesis, thus facilitating experimental testing of evolutionary hypotheses in a phylogenetic context. Ambiguity present in older terminology is clarified; for example, sub-C_4_ clearly defines a phase that is not fully C_4_, as seen in *F. brownii*, instead of the vague term ‘C_4_-like’, which can mean like a C_4_. Sub-C_2_ in turn can encompass a range of incomplete C_2_ states, from weak to almost full C_2_ depending upon the relative strength of the photorespiratory glycine shuttle between M and BS cells. C_3_+ and C_2_+ indicate augmented character states that are largely C_3_ or C_2_ in function, but with additional features that enhance photosynthetic performance.

The C_4_ photosynthetic pathway is hypothesized to have evolved via a series of intermediate steps in which photorespired CO_2_ is trapped and refixed in bundle sheath (BS) cells of C_3_–C_4_ intermediates (Monson *et al.*, 1984; Sage *et al*., 2012; Schlüter and Weber, 2016). The physiology associated with trapping and refixation of photorespired CO_2_ is termed photorespiratory glycine shuttling, or C_2_ photosynthesis, based on the number of carbon atoms shuttled into the BS on the glycine molecule (Sage *et al*., 2014). Initially, C_3_–C_4_ intermediacy was equated with just the phenotype now termed C_2_ photosynthesis because intermediates examined then were largely C_2_ species (Ku *et al.*, 1983; Monson *et al*., 1984; Edwards and Ku, 1987). In the subsequent four decades, additional phenotypes have been identified which are hypothesized to represent a range of evolutionary intermediate stages between the C_3_ and C_4_ character states (reviewed in Stata *et al*., 2019). With improved phylogenetic understanding, it has become possible to hypothesize the position of intermediate character states along putative evolutionary pathways from C_3_ to C_4_ photosynthesis, leading to models of how C_4_ plants evolved (Monson and Rawsthorne, 2000; Sage *et al*., 2012; Heckmann *et al*., 2013; Williams *et al*., 2013; Stata *et al*., 2019). As more character states were identified, however, the nomenclature developed to describe C_3_–C_4_ intermediacy (for example in Edwards and Ku, 1987) has become increasingly deficient. Recently, Stata *et al.* (2019), Adachi *et al.* (2023) and Leung *et al*. (2024) updated the nomenclature of C_3_–C_4_ intermediacy to more completely describe intermediate phenotypes based upon their functional and putative evolutionary context. In summary (Fig. 1), the intermediate character states are described in a progression from an initial C_3_ phenotype to the C_2_ and then C_4_ character states. With this updated system, it is easier to place various intermediate phenotypes within an evolutionary context, which we do in this study for grasses in a C_3_ to C_4_ clade that includes species in the genera *Homolepis* and *Mesosetum* (subtribe Arthropogoninae).

The best studied clade with numerous C_2_ species is the eudicot genus *Flaveria* (Asteraceae). Through numerous physiological, structural and phylogenetic studies, *Flaveria* provides strong support for a hypothesis that C_2_ photosynthesis is a critical intermediate character state in C_4_ evolution (Monson and Rawsthorne, 2000; McKown *et al*., 2005; Adachi *et al*., 2023). Research incorporating phylogenetic, physiological and structural data in the C_4_ eudicot clades *Alternanthera* (Amaranthaceae; Sanchez del Pino *et al*., 2012), *Cleome* (Cleomaceae; Feodorova *et al*., 2010), *Chenopodium* (Chenopodiaceae; Yorimitsu *et al.*, 2019), *Euphorbia* (Euphorbiaceae; Sage *et al*., 2011), *Euploca* (Boraginaceae, formerly *Heliotropium* section *Orthostachys*; Muhaidat *et al*., 2011; Frohlich *et al*., 2022), *Mollugo*/*Hypertelis* (Molluginaceae; Christin *et al*., 2010), Salsoleae (Voznesenskaya *et al.*, 2013) and *Tribulus* (Leung *et al*., 2024) also support the C_2_ phenotype as a transitional state. This frequency of studies of C_2_ and other intermediate states in eudicot clades is not matched in monocots, where only two major clades of *bona fide* C_3_–C_4_ intermediates have been described in the grass genera *Neurachne* and *Alloteropsis* (Hattersley *et al*., 1986; Christin *et al*., 2012*b*; Lundgren *et al*., 2015, 2016; Khoshravesh *et al*., 2020*b*; Pereira *et al*., 2023). A third clade of C_2_ grass species is *Steinchisma*, which lacks a close sister relationship with a C_4_ clade (Alliscioni *et al*., 2003; Zuloaga *et al*., 2018; Acosta *et al*., 2019). The limited number of C_3_–C_4_ intermediates known from the monocots is surprising given that C_4_ grasses and sedges account for a large majority of global C_4_ biomass and represent 75 % of all C_4_ species and about half of the known C_4_ lineages (Still *et al*., 2003; Sage, 2016; Gallaher *et al*., 2022). The relatively low number of reported C_2_ intermediate states in grasses and sedges may be due to incomplete sampling, extinction of C_3_–C_4_ intermediate phenotypes, or the possibility that C_4_ photosynthesis in the monocots evolved in a distinct manner from eudicots, as suggested by a Bayesian model comparing patterns of C_4_ evolution in monocots and eudicots (Williams *et al*., 2013). A distinct evolutionary pathway to C_4_ photosynthesis in monocots could involve intermediate states that are not currently known.

In this study, we use the scheme outlined in Fig. 1 to interpret the evolutionary physiology of species in the grass genus *Homolepis*. *Homolepis* contains five species and is situated within the Arthropogoninae subtribe (Tribe Paspalae) of Neotropical and subtropical grasses, branching in a sister position to a C_4_ clade consisting of *Altoparadisium*, *Arthropogon*, *Keratochlaena*, *Mesosetum* and *Tatianyx* (Zuloaga and Soderstrom, 1985; Kellogg, 2015; Zuloaga *et al*., 2018; Gallaher *et al*., 2022). *Homolepis* species are perennial grasses distributed from subtropical South America to subtropical Mexico, including the Caribbean islands, with a centre of diversity in Brazil (Zuloaga and Soderstrom, 1985; Kellogg, 2015; Gallaher *et al*., 2022). The species *H*. *glutinosa* is proposed to be C_3_ (Giussani *et al*., 2001) while *H. isocalycia* is suggested to be proto-Kranz (Arantes *et al*., 2020) and a third species, *H. aturensis*, has been described as a C_2_ species (Khoshravesh *et al*., 2016). The photosynthetic type of the remaining two species, *H. longispicula* and *H*. *villaricensis*, have not been determined. Given its sister position to a C_4_ clade in the Arthropogoninae, it is possible that *Homolepis* contains numerous C_3_–C_4_ intermediate species, which could represent vestiges of common ancestral states for *Homolepis* and its sister C_4_ clade (Grass Phylogeny Working Group II, 2012). With the definitive identification of proto-Kranz and C_2_ phenotypes in *Homolepis*, this genus could become an important addition to our understanding of how C_4_ photosynthesis evolved in grasses. If additional intermediate character states are present in *Homolepis* species, then *Homolepis* could help address whether C_2_ and C_4_ evolution in grasses proceeded as it did in eudicots.

In this study, we provide a detailed physiological and anatomical assessment of all known *Homolepis* species to contribute to an understanding of C_2_ evolution and thus the early phases of C_4_ evolution in the grasses. We also conducted similar analyses on *Mesosetum loliiforme*, a C_4_ species that is sister to *Homolepis* within the Arthropogoninae phylogeny (Grass Phylogeny Working Group II, 2012). The Grass Phylogeny Working Group suggests using *Mesosetum* as a suitable sister group for *Homolepis* in the study of C_4_ evolution in the Arthropogoninae. A previous anatomical study of vascular tissue indicates that *Mesosetum* expresses the NADP-malic enzyme (NADP-ME) subtype of C_4_ photosynthesis; however, a definitive determination is lacking (Giussani *et al*., 2001). We assessed carbon isotope ratios (δ^13^C) in all *Homolepis* and multiple Arthropogoninae species, and quantified leaf anatomical and cellular features to include immunolocalization of the P-subunit of glycine decarboxylase (GLDP) in *Homolepis* and *M*. *loliiforme*. Gas exchange of intact leaves assessed C_3_, C_2_ and C_4_ photosynthesis in four *Homolepis* species, *M*. *loliiforme* and, for comparison with known C_3_ and C_4_ species, *Phragmites australis* (C_3_) and *Anthephora pubescens* (C_4_). We generated a whole-genome phylogeny of the *Homolepis* genus to place the physiological and anatomical traits in an evolutionary context. Data from the whole-genome phylogenetic network analysis were also used to determine whether hybridization could have facilitated C_2_ or C_4_ trait diversification in this clade. Finally, we confirmed the C_4_ subtype of *Mesosetum* to be NADP-ME through an examination of chloroplast ultrastructure, transcript expression of C_4_ pathway enzymes and assays of core C_4_ enzymes.

## MATERIALS AND METHODS

### Plant material and growth conditions

The following species were collected in the state of Minas Gerais, Brazil, at the indicated coordinates: *Homolepis glutinosa* (21°19ʹ58″S, 44°58ʹ37″W), *H. isocalycia* (19°12’29″S, 44°58’37″W), *H. longispicula* (19°17ʹ11″S, 43°35ʹ18″W) and *Mesosetum loliiforme* (19°7ʹ12″S, 43°35ʹ22″W). *Homolepis aturensis* was obtained from Puerto Viejo, Costa Rica (9°39ʹ27.4″N, 82°45ʹ8.0″W), by Corey Stinson and examined by Khoshravesh *et al.* (2016). This species was remeasured here as part of the comparative analysis within the genus.

Plants were grown at the University of Toronto (Canada) in a greenhouse set to control day/night temperatures of 30 °C/25 °C. Light was provided by natural sunlight supplemented with high-pressure sodium lamps giving ~200 µmol photons m^−2^ s^−1^ at plant height, such that the light intensity on the plants peaked near 1600 µmol m^−2^ s^−1^ on clear days and was ~300 µmol m^−2^ s^−1^ on overcast days. All species were grown in 10-L pots of a sandy loam soil with the exception of *H. longispicula* and *M. loliiforme*, which were grown in a soil mixture of equal parts sand, a commercial clay substrate (Turface MVP; www.turface.com) and a loam topsoil. This soil mixture avoided root rot and improved nutrient uptake in these two species. Plants were watered daily and fertilized weekly using a fertilizer mix prepared by dissolving 30 mL each of two commercial fertilizers (Miracle-Grow 24-10-10 All Purpose Plant Food and 30 mL of Miracle Grow Evergreen Food 30-10-20) in 20 L of water, and then dissolving iron EDDHA, magnesium sulphate and calcium nitrate to give 20 µm iron, 6 mm calcium and 1 mm magnesium sulphate. The grass species *Phragmites australis* (C_3_) and *Anthephora pubescens* (C_4_) were grown for whole-leaf gas exchange analysis of known C_3_ and C_4_ phenotypes, using the same growth and fertilization regime.

### δ^13^C and whole-leaf gas exchange

Leaf δ^13^C was determined on 1–2 mg of dried tissue sampled from herbarium or live specimens of *Homolepis* and *Mesosetum*. Herbarium specimens of *Homolepis* and Arthropogoninae were sampled at the New York Botanical Gardens (see Supplementary Data Table S1 for raw values and voucher information). The δ^13^ C values from 1–2 mg of dried leaf samples were determined by the Stable Isotope Facility at Washington State University (www.isotopes.wsu.edu).

Gas exchange of recent, fully expanded leaves was measured using a Li-6400 gas exchange system (LICOR Biosciences) as previously described (Sage *et al*., 2013). Measurement conditions were 30 ± 1 °C and a leaf vapour pressure deficit of 2 ± 0.4 kPa. For measurement of the response of net CO_2_ assimilation rate (*A*) to intercellular CO_2_ concentration (*C*_i_) at light saturation (*A*/*C*_i_ curves), leaves were first equilibrated at ambient CO_2_ concentration of 400 μmol m^−2^ s^−1^ and at light intensity of 1250 μmol photons m^−2^ s^−1^ for *H. isocalycia* and 1750 μmol photons m^−2^ s^−1^ for the other species. Ambient CO_2_ was reduced in steps to 25 µmol CO_2_ mol^−1^ air for C_2_ and C_4_ species or 50 µmol mol^−1^ for C_3_ species, with measurements made at each step after equilibration. The CO_2_ concentration was then returned to 400 μmol mol^−1^ and increased in steps to 1500 µmol mol^−1^, with measurements made at each step after equilibration. For *A*/*C*_i_ analysis, 4–15 measurements were conducted on each of three to five plants per species. The carboxylation efficiency (CE) was determined as the initial slope of the *A*/*C*_i_ response, with the *x*-intercept of the initial slope used to estimate the CO_2_ compensation point of *A* (*Γ*). The intrinsic water use efficiency (WUE, *A*/*g*_s_) was calculated by dividing *A* by the corresponding stomatal conductance (*g*_s_).

### Leaf anatomy, ultrastructure and immunolocalization

Characteristics of leaf anatomy were determined on leaf sections sampled from the middle of the most recent, fully expanded leaves of *Homolepis* species and *Mesosetum* species using light microscopy (LM), transmission electron microscopy (TEM) and confocal microscopy (CM). Leaf tissue was sampled between 0800 and 1200 h, fixed in 0.5 % glutaraldehyde/4 % paraformaldehyde in sodium cacodylate buffer pH 6.9, and embedded in Araldite and London Resin White (Khoshravesh *et al*., 2017). Herbarium samples of *H*. *villaricensis* were rehydrated and prepared for LM as described by Christin *et al.* (2010). Immunodetection of GLDP using TEM was conducted as described by Khoshravesh *et al.* (2016). Primary rabbit anti-GLDP and secondary antibody (18 nm anti-rabbit IgG gold conjugate; Jackson Immunoresearch) dilutions were 1:100 and 1:20 respectively. Incubation in primary and secondary antibody was for 3 and 1 h respectively. Images for LM were captured with a Zeiss Axiophot equipped with a DP71 Olympus camera and Olympus CellSens image software (Advanced Microscopy Techniques, MA, USA). A Phillips 201 equipped with an Advantage HR camera system (Advanced Microscopy Techniques, Woburn, MA, USA) captured TEM images.

For CM, leaf tissue was fixed in ethanol:acetic acid (3:1) for 24 h, followed by placement in 0.5 m NaOH for 24 h, and then 50 % NaClO until transparent. Leaves were subsequently rinsed in glass-distilled H_2_O (3×), stained overnight in 0.6 % Calcofluor White, and placed in 90 % TDE (2,2ʹ-thiodiethanol; Hasegawa *et al*., 2016). The translucent leaf tissue was mounted in Antifade (Molecular Probes, Eugene, OR, USA) and *z*-stacks were imaged with a Leica TCS SP8 with a Diode 405 laser. In addition, leaves from each species were removed after clearing in 50 % NaClO and used to quantify leaf vein density and internal leaf anatomy.

### Phylogenetic analysis

Extraction, DNA isolation and sequencing of genomic DNA from *Homolepis* species (except *H. aturensis*) and *M. loliiforme* were conducted by the Beijing Genomics Institute (BGI) as described in Supplementary Data Appendix S1. Voucher specimens that provided leaf samples for DNA extraction are listed in online Supplementary Data Table S1. Genomic DNA sequences from *H. aturensis* were determined by the Centre for the Analysis of Genome Evolution and Function at the University of Toronto (cagef.utoronto.ca) via the CTAB method, sheared with Covaris LLC, and used for MGI-PCR-free library prep. Phylogenetic inference on the concatenated super-matrix was conducted using RaxML-ng (v1.2.0; Kozlov *et al*., 2019) with the parameters ‘—all’, used to conduct the search for the best tree and calculation of bootstrap support in one run, and ‘--bs-trees’ to specify 100 bootstrap replicates. The GTR + G + I model was specified for each partition. Coalescent-based phylogenetic inference was performed using ASTRAL (v5.7.1; Zhang *et al*., 2018) on 4189 gene trees generated using FastTree (v2.1.10; Price *et al*., 2010) with parameters nt, gtr and gamma. In addition to standard coalescent-based phylogenetic inference, the SNaQ function in the PhyloNetworks Julia package (Solís-Lemus *et al*., 2017) was used to infer whether hybridization had occurred (see Supplementary Data Appendix S1 for details). Because we found evidence of a hybrid origin for *H. isocalycia*, gene trees were generated for five genes encoding the P, T, L, and H subunits of glycine decarboxylase (GDC); these five genes exist as single genes for all but the L-subunit, where for Poaceae two conserved paralogues exist (Khoshravesh *et al*., 2016). The *Paspalum* genes encoding these subunits are Pavag08G173400 (P), Pavag08G054300 (H), Pavag03G142400 (L), Pavag03G143800 (L) and Pavag06G233900 (T). Reference-based assemblies and alignments for these genes were generated via the same process as the genes used in the species phylogeny, and gene trees were generated using FastTree in the same manner as those used for coalescent-based phylogenetic inference.

### Quantification of mRNA for C_4_ cycle enzymes and enzyme activity assay

We sampled recently mature leaves of *Homolepis* species and *M*. *loliiforme* for examination of mRNA expression for PEP carboxylase (PEPC), NADP-ME, NAD malic enzyme (NAD-ME) and pyruvate, phosphate dikinase (PPDK). Leaves were sampled between 4 and 5 h post-illumination in Biochambers TPC-19 plant growth chambers (www.biochambers.com) set to provide 25 °C night and 30 °C day temperatures, with a 14-h photoperiod and ~1000 µmol photons m^−2^ s^−1^ at the top of the leaf canopy. After assessing RNA integrity with a Bioanalyzer 2100 system (Agilent Technologies, CA, USA), we prepared cDNA libraries following the DNA nanoball protocol (DNB, BGI, https://www.bgi.com/wp-content/uploads/sites/3/2019/04/DNBseq-Rapid-Whole-Genome-Sequencing-Service-Overview.pdf) and sequenced them using PE150. Reads were mapped onto the primary coding sequences of *Paspalum vaginatum* using Hisat2 (v2.2.1; *Kim et al*., 2019) with the same arguments as used above for DNA reads. Transcript reads per million values were calculated by parsing the SAM files generated in this way using a Python script written by co-author M.S.

To complement the mRNA expression data analysis, we assessed whether *Homolepis* species have elevated activities of the C_4_-cycle enzymes PEPC, NADP-ME, NAD-ME and malate dehydrogenase (MDH). Healthy, recently expanded leaves were sampled, extracted and assayed by coupling the oxidation or reduction of NADP(H) or NAD(H) to activity of the C_4_ cycle enzymes (Adachi *et al*., 2023). *Mesosetum* species and *H. longispicula* plants died by the time of assay, so we used the NADP-ME subtype *Gomphrena serrata* (eudicot, Amaranthaceae) as our C_4_ comparison.

### Data analysis

Anatomical features quantified from LM and CM images were obtained from four replicate plants per species with one leaf sampled per replicate. For TEM images, three replicate plants provided images used to quantify GLDP immunolocalization. Cleared leaves were imaged with LM to determine leaf vein density (total vein length per unit leaf area; Ueno *et al*., 2006) and the number of sheath layers surrounding xylem and phloem. Leaf cross-sections were imaged with LM and used to quantify interveinal distance, M-cell number between veins, BS and M-cell area, M and BS tissue area and the percentage of photosynthetic tissue area allocated to the BS (100 % times planar area of BS tissue divided by the sum of planar area of BS tissue and M tissue) on ten images per biological replicate. Paradermal sections were also imaged with LM and CM to analyse the BS cell length and width on 20 cells per plant replicate as described in Khoshravesh *et al.*, (2020b). Leaf cross-sections used for quantification of GLDP immunogold labelling (gold particles) in BS and M cells using TEM were also examined to determine the percentage of BS and M-cell planar area occupied by chloroplasts to include starch, peroxisomes and mitochondria planar area (five cells per cell type per plant replicate). All LM, TEM and CM images were quantified with a Cintiq graphics tablet (Wacom Technology) and ImageJ software (Schneider *et al*., 2012). All replicate measurements within a plant were averaged to give a value for the plant that was then treated as a replicate in the statistical treatments. Differences between means for all parameters obtained from gas exchange, and microscopic images were evaluated using one-way ANOVA with Tukey’s *post hoc* test where the data were normally distributed. Where normality failed, mean differences were tested with a Kruskal–Wallis test followed by Dunn’s *post hoc* test (*P* < 0.05).

Means from the gas exchange and enzyme assays were evaluated using one-way ANOVA with Tukey’s *post hoc* test, with log-transformation to normalize data if needed, or assessed with a Kruskal–Wallis test if non-normality persisted after transformation.

## RESULTS

### Carbon isotope ratios and leaf gas exchange

Leaf δ^13^C values in the five *Homolepis* species ranged between −27 and −32 ‰, which is typical for C_3_ and C_2_ species lacking a moderate to strong C_4_ metabolic cycle (Table 1; Monson *et al*., 1988). Eighteen *Mesosetum* species, three *Arthropogon* species and *Tatianyx arnacites*, all members of the Arthropogoninae, exhibited leaf δ^13^C values between −10 and −14 ‰, which are typical of C_4_ plants.

**Table 1.** Carbon isotope ratios (δ^13^C, parts per thousand) of select *Arthropogon*, *Homolepis*, *Mesosetum* and *Tatianyx* species from the Arthropogoninae subtribe of grasses. See Supplementary Data Table S1 for raw values and collection information for sampled herbarium specimens.

Genus	Species	δ^13^C [mean ± s.e. (*N*)]
*Arthropogon*	*filifolius*	−12.6 ± 0.2 (3)
	*villosus*	−12.6 ± 0.3 (3)
	*xerachne*	−11.3 (1)
		
*Homolepis*	*aturensis*	−30.3 ± 0.5 (6)
	*glutinosa*	−29.5 ± 1.2 (6)
	*isocalycia*	−28.8 ± 0.7 (5)
	*longispicula*	−27.5 ± 0.2 (4)
	*villaricensis*	−31.9 ± 0.5 (4)
*Mesosetum*	*alatum*	−12.0 (1)
	*annuum*	−10.8 (1)
	*arenarium*	−11.5 ± 0.4 (2)
	*blakei*	−12.1 (1)
	*cayense*	−12.2 ± 0.2 (3)
	*chaseae*	−12.2 ± 0.5 (2)
	*chlorostachyum*	−12.3 ± 1.3 (2)
	*comatum*	−13.4 ± 0.2 (2)
	*elytrochaetum*	−11.7 ± 0.2 (2)
	*exaratum*	−11.0 (1)
	*ferrugineum*	−11.7 ± 0.2 (3)
	*filifolium*	−11.9 ± 0.4 (3)
	*gibbosum*	−12.2 ± 0.5 (2)
	*loliiforme*	−13.5 ± 1.2 (5)
	*pappophorum*	−11.0 ± 0.2 (3)
	*pittieri*	−10.3 (1)
	*rottboelliodes*	−12.9 ± 0.4 (4)
	*wrightii*	−10.2 ± 0.3 (2)
*Tatianyx*	*arnacites*	−12.3 ± 0.5 (3)

**Fig. 2.**
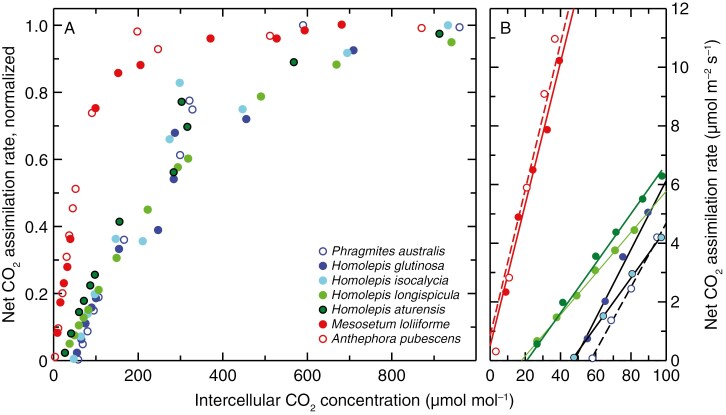
The response of net CO_2_ assimilation rate to intercellular CO_2_ concentration in four *Homolepis* species, a related C_4_ species (*Mesosetum loliiforme*), a known C_3_ (*Phragmites australis*) and a C_4_ species (*Anthephora pubescens*). (A) Species responses normalized to their maximum value at high CO_2_ to better show functional type responses. (B) Initial slope region of the *A*/*C*_i_ response in (A), where *A* is expressed in µmol m^−1^ s^−1^. The plots show responses of individual representative plants. See Table 2 for mean absolute values.

The *A*/*C*_i_ analyses demonstrate a stark difference between the species having C_4_ and non-C_4_ δ^13^C values. The C_4_ species (*A. pubescens*, *M. loliiforme*) exhibited steep initial slopes of the *A*/*C*_i_ response with *A* values rapidly increasing with increasing *C*_i_ below a *C*_i_ of 100 µmol mol^−1^ (Fig. 2A). In the non-C_4_ species, the initial slope of the *A*/*C*_i_ responses was one-third to one-quarter of the value of the C_4_ plants (Fig. 2B), being 0.27–0.29 mol m^−2^ s^−1^ in the two C_4_ species but ranging between 0.07 and 0.1 mol m^−2^ s^−1^ in the non-C_4_ species (Table 2). All the species exhibited similar values of *A* at CO_2_ saturation (*A*_sat_), while the non-C_4_ species showed *A* values at an ambient CO_2_ near 400 µmol mol^−1^, which were about two-thirds the C_4_ values of *A* (Fig. 2; Table 2). Stomatal conductance (*g*_s_) relative to *A* was markedly lower in the C_4_ species, allowing them to have *C*_i_/*C*_a_ ratios about half that of the non-C_4_ species and intrinsic WUE values that were 2–3 times higher. No clear differences in *A*, *A*_sat_ or intrinsic WUE were evident between the species having non-C_4_ values of leaf δ^13^C (Table 2).

**Table 2.** Gas exchange parameters at 30 °C of living species examined in this study and their designated photosynthetic functional type.

	*Phragmites australis* (C3)	*Homolepis glutinosa* (C_3_)	*Homolepis isocalycia* (sub-C2)	*Homolepis longispicula* (full C2)	*Homolepis aturensis* (full C2)	*Mesosetum loliiforme* (C4)	*Anthephora pubescens* (C4)
CO_2_ compensation point (*Γ*, µmol CO_2_ mol^−1^ air)	55.7 ± 3.5^a^	53.7 ± 1.6^a^	44.0 ± 0.9^b^	20.5 ± 1.2^c^	15.7 ± 1.7^c^	1.5 ± 0.7^d^	1.6 ± 1.2^d^
*A*/*C*_i_ response initial slope (mol m^−2^ s^−1^)	0.10 ± 0.002^b^	0.09 ± 0.01^bc^	0.09 ± 0.002^bc^	0.07 ± 0.01^c^	0.08 ± 0.00^bc^	0.27 ± 0.02^a^	0.29 ± 0.01^a^
Net CO_2_ assimilation rate at 400 µmol mol^−1^ (*A*_400_, µmol m^−2^ s^−1^	21.5 ± 0.4^bc^	15.7 ± 1.7bc	18.8 ± 1.5^bc^	16.1 ± 1.1^c^	17.9 ± 0.5^c^	25.2 ± 1.3^ab^	28.6 ± 2.2^a^
Net CO_2_ assimilation rate at high CO_2_ (*A*_sat_, µmol m^−2^ s^−1^)	27.3 ± 0.8^a^	25.4 ± 1.8^a^	26.7 ± 2.7^a^	26.3 ± 2.0^a^	21.5 ± 1.3^a^	26.3 ± 1.9^a^	27.4 ± 2.3^a^
Ratio of *A*_400_ to *A*_sat_	0.79 ± 0.01b	0.62 ± 0.04^c^	0.71 ± 0.07^bc^	0.62 ± 0.02^c^	0.84 ± 0.02^b^	0.96 ± 0.02^ab^	1.05 ± 0.03^a^
Stomatal conductance at 400 ppm (*g*_s_ _400_, mol m^−2^ s^−1^)	0.71 ± 0.03a	0.30 ± 0.05^b^	0.59 ± 0.14^a^	0.32 ± 0.04^b^	0.41 ± 0.06^ab^	0.17 ± 0.01^b^	0.24 ± 0.02^b^
*C*_i_/*C*_a_	0.77 ± 0.03^a^	0.72 ± 0.01^a^	0.77 ± 0.02^a^	0.73 ± 0.01^a^	0.72 ± 0.03^a^	0.34 ± 0.02^b^	0.43 ± 0.02^b^
Intrinsic WUE (*A*_*400*_/*g*_s_ _400_, mmol mol^−1^)	30.4 ± 0.8^d^	52.2 ± 2.1^c^	35.1 ± 4.0^d^	52.7 ± 2.7^c^	45.7 ± 5.8^cd^	148.3 ± 4.8^a^	119.7 ± 5.3^b^

Data are mean ± standard error; *N* = 3–7.

Letters following standard error values indicate statistically different means at *P* < 0.05 via one-way ANOVA with Tukey’s *post hoc* test.

*A*, net CO_2_ assimilation rate; *C*_a_, ambient CO_2_ concentration; *C*_i_, intercellular CO_2_ concentration; WUE, water use efficiency. Note that the initial slope of the *A*/*Ci* response is considered carboxylation efficiency (CE).

Examination of the photosynthetic CO_2_ compensation point of *A* (*Γ*) showed *H. glutinosa* to have a C_3_-like *Γ* value equivalent to that of the known C_3_ species *P. australis*, while the C_4_ species exhibited low *Γ* values near 1.5 µmol mol^−1^, as is typical for C_4_ species (Table 2). *Homolepis aturensis* and *H. longispicula* exhibited *Γ* values between 15 and 21 µmol mol^−1^, respectively, which are typical of species with well-developed C_2_ photosynthesis, while *H. isocalycia* exhibited a *Γ* value of 44 µmol mol^−1^, which is statistically different from the C_3_ and C_2_ values (Table 2).

### Leaf morphology, anatomy and immunolocalization of GLDP

**Fig. 3.**
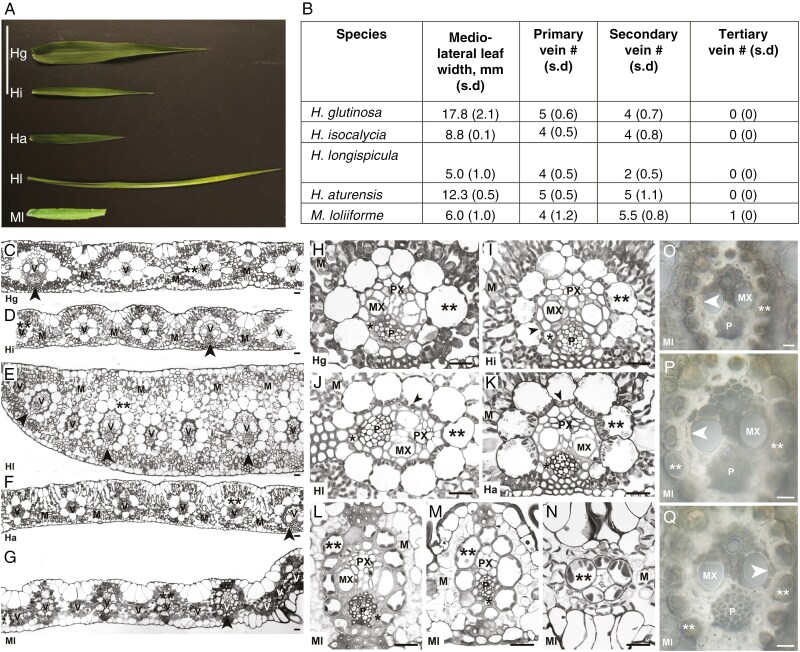
Leaf morphology and internal anatomy of *Homolepis* species and *Mesosetum loliiforme*. (A) External leaf morphology. (B) Mean leaf width and number of primary veins, secondary vein number between lateral veins, and tertiary vein number between secondary veins. (C–Q) internal leaf anatomy; (H–L) primary vein anatomy. (M, N) Primary, secondary and tertiary vein anatomy of *M*. *loliiforme*. (O–Q) Hand sections of *M*. *loliiforme* illustrating inner and outer sheaths of primary veins. Scale bars: (A) = 5 cm; (C–Q) = 20 μm. M, mesophyll; MX, metaxylem; P, phloem; PX, protoxylem; V, vascular tissue. Single asterisk, inner sheath; double asterisks, outer sheath; black arrowhead (C–G), primary vein; black arrowhead (H–N) highlights organelles adjacent to vascular tissue; white arrowhead (O–Q) marks inner sheath cells adjacent to metaxylem at low (O) and high (P, Q) magnification. Hg, *H*. *glutinosa* (C_3_); Hi, *H*. *isocalycia* (sub-C_2_); Hl, *H*. *longispicula* (C_2_); Ha, *H*. *aturensis* (C_2_); Ml, *M*. *loliiforme* (C_4_).

**Fig. 4.**
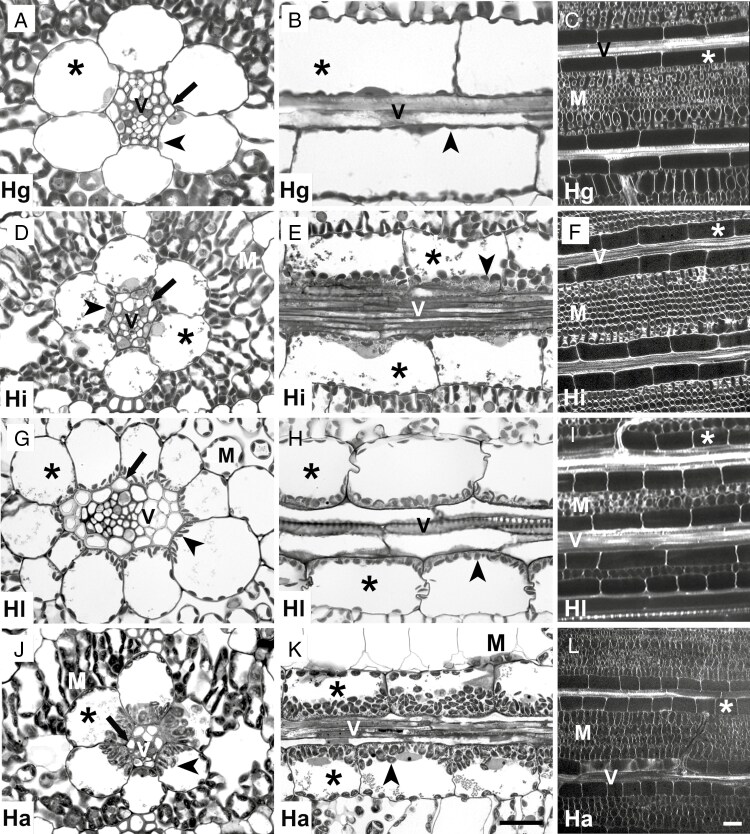
Light and confocal micrographs illustrating outer sheath cells from secondary veins of four *Homolepis* species. (A, D, G, J) Transverse and (B, E, H, K) paradermal sections imaged with a light microscope. (C, F, I, L) Paradermal views imaged with a confocal microscope. Scale bar = 20 μm (light micrographs) or 40 μm (confocal images). V, vascular tissue; asterisk, outer sheath; arrowheads show organelles adjacent to vascular tissues; arrows mark inner sheath. Hg, *H*. *glutinosa* (C_3_); Hi, *H*. *isocalycia* (sub-C_2_); Hl, *H*. *longispicula* (C_2_); Ha, *H*. *aturensis* (C_2_).

The morphology of leaves of *Homolepis* and *M*. *loliiforme* are different from one another in length and medio-lateral width, with *H. glutinosa* and *H. longispicula* developing the widest and narrowest leaves, respectively (Fig. 3A, B). The mesophyll tissue of all species consists of three layers of M cells between the adaxial and abaxial epidermis except for *H*. *longispicula*, which has approximately ten M-cell layers (Fig. 3C–G; Supplementary Data Fig. S1). The M-cell number between veins and the interveinal distance are reduced at lower *Γ* in the *Homolepis* species and *M*. *loliiforme* (Table 3). Notably, the number of M cells between veins is two in *H*. *longispicula* and *M*. *loliiforme*, which conforms to the two M cells between veins observed in many C_4_ clades (Table 3; Hattersley and Watson, 1975; Dengler and Nelson, 1999). The vascular anatomy of *Homolepis* and *M*. *loliiforme* is typical of grasses, where primary veins contain proto- and metaxylem and secondary veins develop only protoxylem (Fig. 3C–M; Fig. 4A, D, G, J; Supplementary Data Fig. S1; Renvoize, 1987). Vein density is greater in *H. isocalycia*, *H. longispicula* and *H. aturensis* than in *H. glutinosa* and is highest in *M*. *loliiforme* (Fig. 3C–G; Table 3). In addition to the primary and secondary veins, the C_4_  *M*. *loliiforme* develops a single tertiary vein between primary and secondary veins (Fig. 3B, G, N) as reported for *Alloteropsis semialata* (Lundgren *et al.*, 2019).

**Table 3.** Anatomical parameters of bundle sheath (BS) and mesophyll (M) cells in *Homolepis* and *Mesosetum* leaves sampled from live specimens, measured from images made using light microscopy (LM) or confocal microscopy (CM).

Parameter	*Homolepis glutinosa* (C3)	*Homolepis isocalycia* (sub-C_2_)	*Homolepis longispicula* (full C2)	*Homolepis aturensis* (full C2)	*Mesosetum loliiforme* (C4)
M/BS tissue area ratio (LM)	2.7 ± 0.1^a^	2.7 ± 0.1^a^	2.0 ± 0.2^b^	2.2 ± 0.1^ab^*	1.8 ± 0.1^b^
Percentage of photosynthetic tissue as BS (LM)^1^	27.4 ± 0.9^b^	27.4 ± 0.8^b^	33.2 ± 1.9^a^	30.9 ± 0.5^ab^*	35.4 ± 1.3^a^
Number of M cells between veins (LM)	8.3 ± 0.5^a^	7.1 ± 0.3^a^	2.2 ± 0.3^c^	5.6 ± 0.2^b^	2.1 ± 0.1^c^
M cell size, transverse planar area, LM (µm^2^)	162 ± 18^ab^	185 ± 18^ab^	296 ± 58^a^	235 ± 27^ab^	112 ± 12^b^
Interveinal distance, LM (µm)	156 ± 13^a^	104 ± 6^b^	31 ± 7^c^	76 ± 5^b^	33 ± 3^c^
Vein density, LM (number µm^−2^)	3.7 ± 0.1^c^	5.5 ± 0.1^b^	6.1 ± 0.8^b^	6.4 ± 0.3^b^	10.8 ± 0.4^a^
BS cell volume, CM (µm^3^)	78.2 ± 7.8^a^	41.1 ± 4.5^bc^	49.3 ± 9.3^b^	21.9 ± 0.9^cd^	5.4 ± 0.2^d^
BS cell length in paradermal view, CM (µm)	92.6 ± 4.4^a^	75.6 ± 5.0^b^	80.4 ± 0.5^ab^	52.2 ± 1.5^c^	31.1 ± 1.8^d^
BS cell width in paradermal view, CM (µm)	40.9 ± 3.1^a^	33.1 ± 0.9^ab^	37.2 ± 3.3^ab^	29.9 ± 0.2^b^	18.2 ± 1.1^c^
BS cell length/width, CM (*P* = 0.11)	2.3 ± 0.3	2.3 ± 0.1	2.2 ± 0.2	1.8 ± 0.04	1.7 ± 0.2
BS cell area in paradermal view, LM (µm^2^)	3634 ± 134^a^	1741 ± 68^b^	3044 ± 343^a^	1386 ± 73^bc^	1081 ± 106^c^
BS cell area in transverse view, LM (µm^2^)	771 ± 72^b^	460 ± 18^c^	871 ± 5^a^	771 ± 25^b^	471 ± 8^c^

Data are mean ± standard error; *N* = 3–4.

Letters following standard error values indicate statistically different means at *P* < 0.05 via one-way ANOVA with Tukey’s *post hoc* test.

*The *H. aturensis* M/BS ratio and percentage of photosynthetic tissue in the BS were significantly different (*P* < 0.02) from the corresponding means in *H. glutinosa* via Student’s *t*-test.

^1^The percentage of photosynthetic tissue as BS was calculated as 100 % × the planar cross-sectional area of BS tissue divided by the sum of the planar area of BS tissue and M tissue in a light micrograph of a leaf cross-section.

**Fig. 5.**
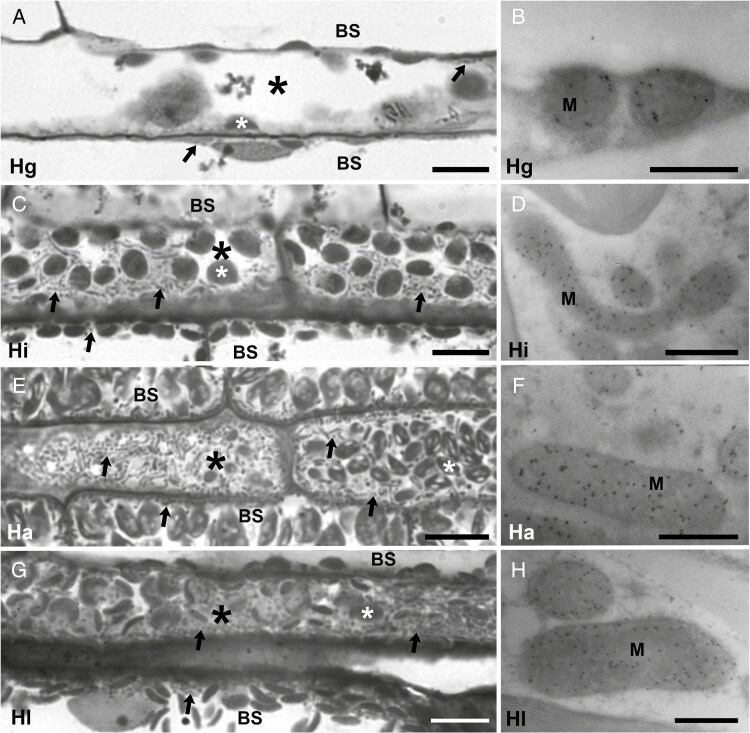
Light and transmission electron micrographs of bundle sheath cells of four *Homolepis* species. In panels (A), (C), (E) and (G), paradermal sections illustrate centripetally positioned organelles in BS cells. Panels (B), (D), (F) and (H) show immunogold labelling of glycine decarboxylase P-subunit (black dots) in BS mitochondria. Scale bar (A, C, E, G) = 10 μm; (B, D, F, H) = 500 nm. BS, bundle sheath cells; M, mitochondria. White asterisk labels chloroplasts; arrow highlights mitochondria. Black asterisk marks bundle sheath cells situated above adaxial mestome sheath cells. Hg, *H*. *glutinosa* (C_3_); Hi, *H*. *isocalycia* (sub-C_2_); Hl, *H*. *longispicula* (C_2_); Ha, *H*. *aturensis* (C_2_).

The primary and secondary veins of leaves from the *Homolepis* species and *M*. *loliiforme* have an inner sheath and outer sheath surrounding the vascular bundles (Figs 3C–M, O–Q and 4A, D, G, J; Supplementary Data Fig. S1). The inner sheath of primary veins is continuous in the *Homolepis* species (Fig. 3C–K; Supplementary Data Fig. S1). Our hand-sections of cleared leaves showed that the inner sheath of primary veins in *M*. *loliiforme* is usually continuous (Fig. 3O–Q) but showed occasional discontinuity opposite the metaxylem elements (Fig. 3L). The outer sheath cells in the *Homolepis* species are enlarged in planar cross-section (Figs 3C–K and 4A, D, G, J; Supplementary Data Fig. S1) compared with what is reported for rice and other C_3_ grasses (Christin *et al*., 2013; Wang *et al*., 2017). In the C_2_  *Homolepis* species and the C_4_  *M. loliiforme*, outer sheath cells are qualitatively enriched with organelles to include chloroplasts (Figs 3I–M, 4D, E, G, H, J, K and 5C, E, G; Supplementary Data Figs S2B–D and S4A); by contrast, no chloroplasts are evident in inner sheath cells of these species (Figs 3I–M and 4D, G, J; Supplementary Data Fig. S2B–D). From these results, we conclude that the outer sheath of C_2_  *Homolepis* species is where photorespired CO_2_ is trapped and refixed, while in *M*. *loliiforme* the outer sheath is the site of C_4_-acid decarboxylation and CO_2_ concentration. Based on prior criteria (Brown, 1975; Dengler *et al.*, 1985), we conclude the outer sheath is equivalent to parenchymatous BS in *Homolepis* and *M*. *loliiforme*, while the inner sheath corresponds to the mestome sheath.

Anatomical assessments of planar leaf sections of *Homolepis* and *M*. *loliiforme* show that the M/BS area ratio was ~20 % lower in *H. longispicula*, *H. aturensis* and *M. loliiforme* than in the C_3_ plant *H. glutinosa*, while the proportion of photosynthetic tissue area allocated to the BS was 10–20 % higher in these three species relative to *H. glutinosa* (Table 3). The volume, length, width and paradermal area of individual BS cells generally decline as *Γ* declines in the *Homolepis* species and *M*. *loliiforme*, except for *H. longispicula*, which has length, width and planar areas in transverse and paradermal section that are similar to the corresponding values in *H. glutinosa* (Figs 3 and 4; Table 3).

**Fig. 6.**
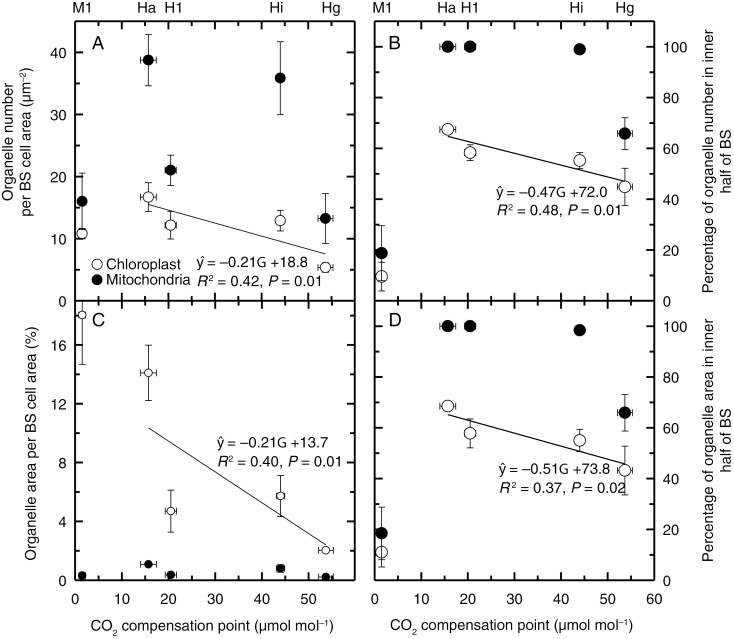
Chloroplast and mitochondria parameters of bundle sheath cells of *Homolepis* species and *Mesosetum loliiforme* expressed as a function of the corresponding CO_2_ compensation point of photosynthesis (*Γ*). The *Γ* values corresponding to the species are indicated by the abbreviations above panels (A) and (B): Hg, *H*. *glutinosa* (C_3_); Hi, *H*. *isocalycia* (sub-C_2_); Hl, *H*. *longispicula* (full C_2_); Ha, *H*. *aturensis* (full C_2_); Ml, *M*. *loliiforme* (C_4_). Significant regressions for the chloroplast parameters from the four *Homolepis* species are shown. Regressions between the mitochondrial data lacked a significant slope and are not shown. Data points show mean ± standard error, *N* = 3. Organelles per BS cell area refers to the number of chloroplasts or mitochondria in the BS cells, divided by the corresponding BS cell area in the measured image. The unit µm^−2^ is short for organelle number per µm^−2^ of chloroplast planar area. The percentage of organelles in the inner half of the BS is the number of chloroplasts or mitochondria in the inner half of a BS cell in planar view, divided by the total number of the corresponding organelle type in the BS cell × 100 %. Organelle area is the measured planar area of chloroplasts or mitochondria within a BS cell divided by the planar area of that BS cell × 100 %, while the percentage organelle area in the inner half of the BS is the sum of the chloroplast or mitochondria area in the inner half of the BS divided by the respective sum of the chloroplast or mitochondrial area in the same BS cell × 100 %.

Chloroplast numbers per BS cell area in planar view were on average two to three times greater in *H. isocalycia*, *H. longispicula*, *H. aturensis* and *M. loliiforme* than in *H. glutinosa*, and a regression analysis between *Γ* and chloroplast numbers per BS area indicated that these differences were significant (Fig. 6A); however, significant differences via a Tukey’s test were only detected between *H. glutinosa*, and *H. aturensis and M. loliiforme* (Table 4). The planar area of individual BS chloroplasts was similar between the *Homolepis* species but four times greater in *M. loliiforme* than in *H. glutinosa*, demonstrating that the C_4_ plant has substantially larger BS chloroplasts. *Homolepis aturensis* and *M. loliiforme* exhibited seven to nine times greater percentage chloroplast planar area per BS cell planar area than *H. glutinosa* (Table 4), with *H. isocalycia* and *H. longispicula* having intermediate values (as indicated by the regression analysis in Fig. 6C; Table 4).

**Table 4.** Chloroplast and peroxisome parameters in leaves sampled from live *Homolepis* and *Mesosetum* species, measured from planar TEM images of transverse (= cross) sections.

Parameter	*Homolepis glutinosa* (C3)	*Homolepis isocalycia* (sub-C2)	*Homolepis longispicula* (full C2)	*Homolepis aturensis* (full C2)	*Mesosetum loliiforme* (C4)
Chloroplast parameters					
Chloroplast number per BS-cell area × 1000 (µm^−2^)	5.4 ± 0.8^bc^	12.9 ± 1.7^ab^	12.2 ± 2.2^ab^	16.7 ± 2.3^a^	10.8 ± 0.8^ab^
BS chloroplast size, planar area, µm^−2^	4.2 ± 0.8^b^	4.3 ± 0.5^b^	3.7 ± 0.6^b^	8.7 ± 1.6^b^	17.3 ± 3.0^a^
Percentage chloroplast area per BS-cell area	2.0 ± 0.12^c^	5.7 ± 1.4^bc^	4.7 ± 1.4^c^	14.1 ± 1.9^ab^	18.0 ± 3.4^a^
Percentage of chloroplast number in inner half of BS	44.8 ± 7.3^b^	55.2 ± 3.2^ab^	58.3 ± 3.1^ab^	67.4 ± 1.7^a^	9.6 ± 5.7^c^
Percentage of chloroplast area in inner half of BS	40.7 ± 5.8^b^	53.7 ± 3.8^ab^	58.0 ± 5.6^ab^	67.9 ± 0.2^a^	10.2 ± 5.1^c^
Chloroplast number per M-cell area × 1000 (µm^−2^) (*P* = 0.18)	40.7 ± 5.5	44.8 ± 3.4	28.0 ± 4.9	34.2 ± 1.9	30.9 ± 7.4
M-cell chloroplast size, planar area (µm^2^)	10.4 ± 1.4^a^	7.8 ± 1.1^ab^	3.7 ± 0.1^b^	9.4 ± 0.8^a^	5.5 ± 0.7^b^
Percentage chloroplast area per M-cell area	42.0 ± 8.7^a^	33.3 ± 6.4^ab^	9.6 ± 1.7^c^	29.7 ± 3.4^ab^	14.9 ± 2.1^bc^
Percentage leaf chloroplast number in BS tissue	5.2 ± 1.2^b^	10.1 ± 0.6^ab^	18.4 ± 2.8^a^	17.6 ± 2.6^a^	16.6 ± 2.1^a^
Percentage leaf chloroplast area in BS tissue	2.0 ± 0.3^d^	6.2 ± 0.4^c^	18.9 ± 2.1^ab^	17.5 ± 3.3^b^	39.0 ± 6.4^a^
Peroxisome parameters					
Peroxisome number per BS-cell area × 1000 (µm^−2^) (*P* = 0.13)	2.1 ± 0.8	7.9 ± 2.6	5.5 ± 0.7	10.9 ± 2.0	5.4 ± 3.4
BS peroxisome size, planar area µm^−2^ (*P* = 0.11)	0.19 ± 0.04	0.18 ± 0.03	0.19 ± 0.06	0.28 ± 0.01	0.11 ± 0.02
Percentage peroxisome area per BS-cell area (*P* = 0.08)	0.04 ± 0.02	0.15 ± 0.07	0.10 ± 0.04	0.32 ± 0.11	0.08 ± 0.06
Peroxisome number per M-cell area × 1000 (µm^−2^)	30.1 ± 5.8^a^	29.8 ± 1.5^a^	9.3 ± 2.6^b^	17.4 ± 2.7^ab^	22.6 ± 2.9^ab^
M-cell peroxisome size, planar area (µm^2^)	0.33 ± 0.06^a^	0.19 ± 0.03^ab^	0.12 ± 0.02^b^	0.20 ± 0.05^ab^	0.12 ± 0.03^b^
Percentage peroxisome area in M-cell area	0.86 ± 0.07^a^	0.58 ± 0.09^b^	0.11 ± 0.04^c^	0.29 ± 0.01^c^	0.26 ± 0.01^c^
Percentage of leaf peroxisome number in BS tissue	2.5 ± 0.9^b^	9.4 ± 3.1^ab^	24.9 ± 5.0^a^	22.5 ± 6.5^a^	10.5 ± 6.1^ab^
Percentage of leaf peroxisome area in BS tissue	1.8 ± 0.7^b^	8.6 ± 3.5^b^	30.7 ± 3.5^a^	31.1 ± 7.3^a^	12.0 ± 8.9^ab^

Data are mean ± standard error, *N* = 3.

Letters following standard error values indicate statistically different means at *P* < 0.05 via one-way ANOVA with Tukey’s *post hoc* test.

The BS chloroplasts of *M*. *loliiforme* are positioned centrifugally along the outer cell wall of the BS, while in the *Homolepis* species chloroplasts tend to occur in both halves of the BS, with a modest increase in chloroplast number and area evident in the inner half of the BS cells as *Γ* declines (as indicated by the linear regression in Fig. 6B, D; see also Figs 3H–K and 4A, B, D, E, G, H, J, K, and Supplementary Data Figs S2A–D and S4A). In *H. villaricensis*, cross-sectional images prepared using rehydrated herbarium specimens resemble the BS cell images from *H. glutinosa* in that the BS cells are largely empty with no consistent centripetal aggregation of organelles (Figs 3C, H and 4A compared with Supplementary Data Fig. S1). In contrast to patterns in the BS, we detected no difference in chloroplast number per M-cell area between the study species. *Mesosetum loliiforme* and *H. longispicula* had smaller M-cell chloroplasts than the other *Homolepis* species, leading to chloroplast area per M-cell area values that are one-third to one-quarter of those observed for *H. glutinosa* (Table 4).

The BS chloroplasts of *M*. *loliiforme* contain fewer, smaller grana than M chloroplasts (Supplementary Data Fig. 4 B, E), as reported for BS chloroplasts in NADP-ME C_4_ subtypes (Dengler and Nelson, 1999). The BS cell wall of *M*. *loliiforme* is qualitatively thicker than that of *Homolepis* species (Supplementary Data Fig. S2A–D versus Supplementary Data Fig. S4A) and contains an electron-dense suberin lamella (Supplementary Data Figs S4A and S5I, J), present in the BS and mestome sheath of numerous grasses (Hattersley and Browning, 1981; Eastman *et al*., 1988; Dengler and Nelson, 1999; Ueno and Sentoku, 2006). A second *Mesosetum* species, *M*. *ferrugineum* has BS cell wall characteristics similar to those of *M*. *loliiforme* (Supplementary Data Fig. S5K). We observed no evidence of a suberin band in the BS cell wall of the *Homolepis* species (Supplementary Data Fig. S5A–H).

Relative to the C_3_  *H. glutinosa*, BS mitochondria number per BS cell area is elevated in *H. isocalycia* and *H. aturensis* (Fig. 5A versus Figs 5C, E, G and 6A; Table 5; Supplementary Data Fig. S2). In *H. glutinosa*, two-thirds of the mitochondria observed in the BS occur within the inner half, while in the other three *Homolepis* species 99 % or more of the BS mitochondria are present in the inner half of the BS cells (Fig. 6B, D; Table 5). *Mesosetum loliiforme* had low values of mitochondria in the BS, similar to values in *H. glutinosa*, and these mitochondria were largely in the outer BS, in striking contrast to the C_2_ pattern (Table 5). In the M cells, Pearson correlation analysis supports an interpretation that reduction in mitochondria numbers per M-cell area occurs as *Γ* declines, leading to greater mitochondrial area per M-cell area in *H. glutinosa* than observed in *H. longispicula*, *H. aturensis* and *M. loliiforme* (Table 5).

**Table 5.** Mitochondria and glycine decarboxylase parameters in leaves sampled from living *Homolepis* and *Mesosetum* species, measured from planar TEM images of transverse (=cross) sections.

Parameter	*Homolepis glutinosa* (C_3_)	*Homolepis isocalycia* (weak C_2_)	*Homolepis longispicula* (full C_2_)	*Homolepis aturensis* (full C_2_)	*Mesosetum loliiforme* (C_4_)
Mitochondria parameters					
Mitochondria number per BS-cell area × 1000 (µm^−2^)	13.2 ± 4^c^	35.9 ± 5.9^ab^	21.0 ± 2.4^abc^	38.8 ± 4.1^a^	16.0 ± 4.6^bc^
BS mitochondria size, planar area, (µm^−2^) (NS, *P* = 0.22)	0.16 ± 0.03	0.23 ± 0.07	0.17 ± 0.03	0.29 ± 0.03	0.20 ± 0.01
Percentage mitochondria area per BS-cell area	0.22 ± 0.09^b^	0.80 ± 0.26^ab^	0.36 ± 0.11^b^	1.08 ± 0.05^a^	0.31 ± 0.09^b^
Percentage of mitochondria number in inner half of BS	66 ± 6^b^	99 ± 1^a^	100 ± 0^a^	100 ± 0^a^	19 ± 11^c^
Percentage of mitochondria area in inner half of BS	66 ± 7^b^	98 ± 2^a^	100 ± 0^a^	100 ± 0^a^	18 ± 10^c^
Mitochondria number per M cell area × 1000 (µm^−2^) (*P* = 0.036)^1^	68 ± 23	51 ± 8	15 ± 3	29 ± 5	15 ± 5
M-cell mitochondria size, planar area (µm^2^)	0.31 ± 0.02^a^	0.27 ± 0.04^ab^	0.12 ± 0.02^b^	0.24 ± 0.05^ab^	0.19 ± 0.02^ab^
Percentage mitochondria per M-cell area	2.0 ± 0.8^a^	1.4 ± 0.1^a^	0.2 ± 0.0^c^	0.7 ± 0.1^ab^	0.2 ± 0.1^bc^
Percentage of leaf mitochondria number in BS tissue	7.5 ± 1.5^b^	22.5 ± 5.4^ab^	42.6 ± 4.1^a^	37.7 ± 6.1^a^	37.5 ± 10.9^a^
Percentage of leaf mitochondria area in BS tissue	4.0 ± 0.6^b^	18.2 ± 3.9^ab^	52.5 ± 8.4^a^	42.6 ± 6.6^a^	41.9 ± 13.9^a^
GLDP immunolocalization					
GLDP immunogold label per BS-cell area × 1000 (µm^−2^)	91 ± 0.04^c^	660 ± 163^ab^	322 ± 110^bc^	1029 ± 164^a^	129 ± 57^bc^
GLDP immunogold label per BS mitochondria area (µm^−2^)	24 ± 5^b^	78 ± 20^ab^	60 ± 15^ab^	110 ± 8^a^	28 ± 7^b^
GLDP immunogold label per M-cell area, × 1000 (µm^−2^)	1490 ± 528^a^	524 ± 134^ab^	22 ± 3^b^	38 ± 6^b^	24 ± 9^b^
GLDP immunogold label per M mitochondria area (µm^−2^)	82 ± 9^a^	44 ± 10^ab^	15 ± 1^b^	7 ± 1^b^	12 ± 4^b^
Percentage of leaf immunogold label in BS tissue	2.1 ± 0.6^c^	34.4 ± 6.8^b^	86.5 ± 3.6^a^	91.6 ± 2.4^a^	70.0 ± 12.1^a^

Data are mean ± standard error, *N* = 3 plants.

Letters following standard error values indicate statistically different means at *P* < 0.05 via one-way ANOVA with Tukey’s *post hoc* test.

^1^Pearson correlation analysis between *Γ* and mitochondria number per M-cell area showed a significant correlation (correlation coefficient 0.73, *P* = 0.002), indicating the mitochondria numbers per M-cell area in *H. longispicula*, *H. aturensis* and *M. loliiforme* are significantly lower than in *H. glutinosa*.

**Fig. 7.**
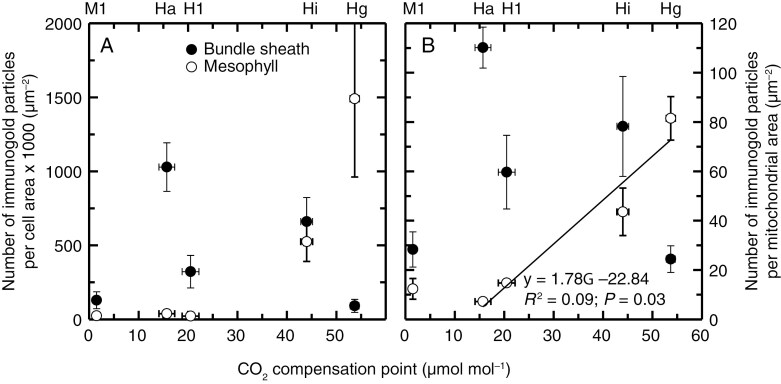
Relationship between photosynthetic CO_2_ compensation point (*Γ*) and the number of immunogold labels for GLDP in four *Homolepis* species or *M. loliiforme*, expressed as immunodot density per cell area (A) or per mitochondrial area (B) in bundle sheath (filled circles) and mesophyll (open circles) tissue. Data points show means ± standard error, *N* = 3. The unit µm^−2^ is short for immunogold particle number per µm^−2^ of planar cell area × 100 % in panel (A) and immunogold particle number per µm^−2^ of mitochondrial area × 100 % in panel (B). The *Γ* values corresponding to the species are indicated by the abbreviations above panel (A) and (B): Hg, *H*. *glutinosa* (C_3_); Hi, *H*. *isocalycia* (sub-C_2_); Hl, *H*. *longispicula* (C_2_); Ha, *H*. *aturensis* (C_2_); Ml, *M*. *loliiforme* (C_4_). The significant regression shown in panel (B) is for the four *Homolepis* species only.

The density of GLDP immunolabelling as determined by gold particle number per BS cell area was greater on average in the *Homolepis* species with lower *Γ* relative to *H. glutinosa* and *M. loliiforme* (Table 5; Fig. 7A; Supplementary Data Fig. S2E–H), while in the M cells the gold particle density declined in number as *Γ* declined, being almost absent in *H. aturensis* and *H. longispicula* (Fig. 7A; Table 5; Supplementary Data Fig. S3B, D, F, H). Within individual BS mitochondria, the density of gold particles was on average higher in *H. aturensis*, *H. longispicula* and *H. isocalycia* than in *H. glutinosa* and the C_4_  *M. loliiforme*, while in the M cells the GLDP density per mitochondria area declined with lower *Γ* (Fig. 7B; Table 5).

**Fig. 8.**
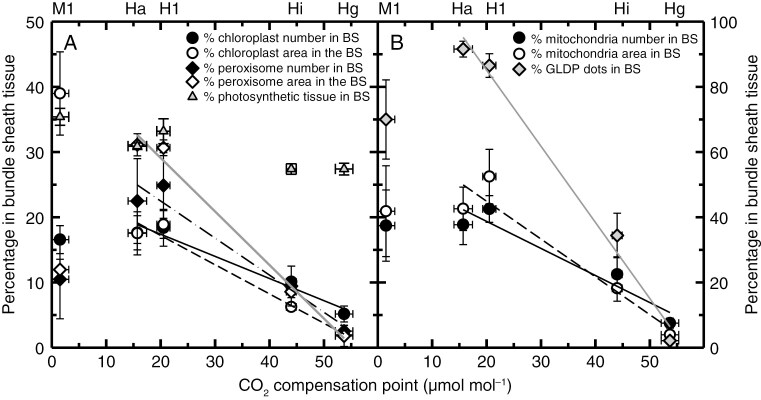
Relationship between photosynthetic CO_2_ compensation point (*Γ*) and relative allocation to the BS tissue of chloroplasts and peroxisomes (A), and mitochondria and immunogold particles binding to the P-subunit of glycine decarboxylase (GLDP dots; B) of four species of *Homolepis* and *M. loliiforme.* Data points show mean ± standard error, *N* = 3. The *Γ* values correspond to the species indicated by abbreviations above panels (A) and (B): Hg, *H*. *glutinosa* (C_3_); Hi, *H*. *isocalycia* (sub-C_2_); Hl, *H*. *longispicula* (C_2_); Ha, *H*. *aturensis* (C_2_); Ml, *M*. *loliiforme* (C_4_). The term % in bundle sheath tissue refers to the percentage of chloroplast or peroxisome numbers (or area) quantified in individual BS cells, divided by the total number of chloroplast or peroxisomes numbers (or planar area) in BS plus mesophyll tissue. Significant regressions are shown for the responses of the four *Homolepis* species only. Panel (A) regressions: *ŷ* = 24.3–0.34*Γ* for percentage chloroplast number in BS (*R*^2^ = 0.95, *P* = 0.02); *ŷ* = 26.2–0.45*Γ* for percentage chloroplast area in BS (*R*^2^ = 0.96, *P* = 0.02); *ŷ* = 33.9–0.57*Γ* for percentage peroxisome number in BS (*R*^2^ = 0.94, *P* = 0.02); *ŷ* = 26.2–0.45*Γ* for percentage peroxisome area in BS (*R*^2^ = 0.96, *P* = 0.005). In panel (B), *ŷ* = 55.4–0.83*Γ* for percentage mitochondria number in BS (*R*^2^ = 0.88, *P* = 0.04); *ŷ* = 68.2–0.1.16*Γ* for percentage mitochondria area in BS (*R*^2^ = 0.87, *P* = 0.04); *ŷ* = 132–2.4*Γ* for percentage GLDP immunogold particles in the BS (*R*^2^ = 0.98, *P* < 0.006).

Figure 8 shows the percentages of chloroplasts, peroxisomes, mitochondria and GLDP immunogold label in the BS tissue as well as the percentage of photosynthetic tissue in the BS in response to declining *Γ*. The percentage of photosynthetic tissue in BS is calculated as 100 % times the planar area of BS tissues divided by the sum of the planar area of BS tissue and M tissue in a light micrograph of a leaf cross-section. While photosynthetic tissue area in the BS shows a slight increase at the lowest *Γ* values (Fig. 8A; see also Table 5 for ANOVA results), large increases in the BS fraction of chloroplast area and numbers, mitochondrial area and numbers, and GLDP immunogold label were evident as *Γ* declined in the four *Homolepis* species. For example, the percentage of GLDP immunogold label in the BS rose as *Γ* declined, from below 10 % in *H. glutinosa* to over 80 % in *H. longispicula* and *H. aturensis* (Fig. 8B; Table 5). The percentage of the leaf peroxisome number and area in the BS was also greater in the *Homolepis* species with reduced *Γ* (Fig. 8A; Table 4). This reflects a combination of an increase in peroxisome number or size in the BS, and/or reduced numbers in the M cells (Table 4).

### Principal component analysis

A principal component analysis of all measurements demonstrated clustering of replicates within individual species, with the species clusters segregating along the first and second principal components (Supplementary Data Fig. S6A). The species with intermediate *Γ* values, including *H. isocalycia*, clustered between the C_3_  *H. glutinosa* and the C_4_  *M. loliiforme* on PC1, and below the C_3_ and C_4_ species on PC2 (Supplementary Data Fig. S6A). Leaf mesophyll and gas exchange parameters largely explained segregation of the species along PC1, while BS properties largely explained separation along PC2 (Supplementary Data Fig. S6B).

### Expression of genes encoding core C_4_ cycle enzymes and enzyme activity

Expression analysis of genes encoding the C_4_ cycle enzymes PEPC, NADP-ME and NAD-ME identified six genes in *Paspalum vaginatum* encoding PEPC and NADP-ME, and two each encoding NAD-ME and PPDK. Of these, three were identified as encoding high expression of PEPC and one gene each encoded high expression of NADP-ME and PPDK in *M. loliiforme* (Supplementary Data Table S2). The enhanced expression values indicate these are the genes expressed in the C_4_ metabolic cycle of *M. loliiforme*. In *H. glutinosa* and *H. isocalycia*, little enhanced expression of any of the PEPC, NAD-ME or PPDK genes was observed. Expression levels of NADP-ME transcripts were higher in *H. glutinosa* than *H. isocalycia*, but these were from NADP-ME genes that did not encode the C_4_ isoform in *M. loliiforme.*

The mean activity of PEPC, NADP-ME and NAD-ME was low in all *Homolepis* species compared with the C_4_ species *Gomphrena serrata* (Supplementary Data Table S3) and other C_4_ NADP-ME grasses (Gutierrez *et al*., 1974). There were no significant differences in activity of these enzymes between *H. aturensis*, *H. isocalycia* and *H. glutinosa* (Supplementary Data Table S3).

### Phylogenetic analyses

**Fig. 9.**
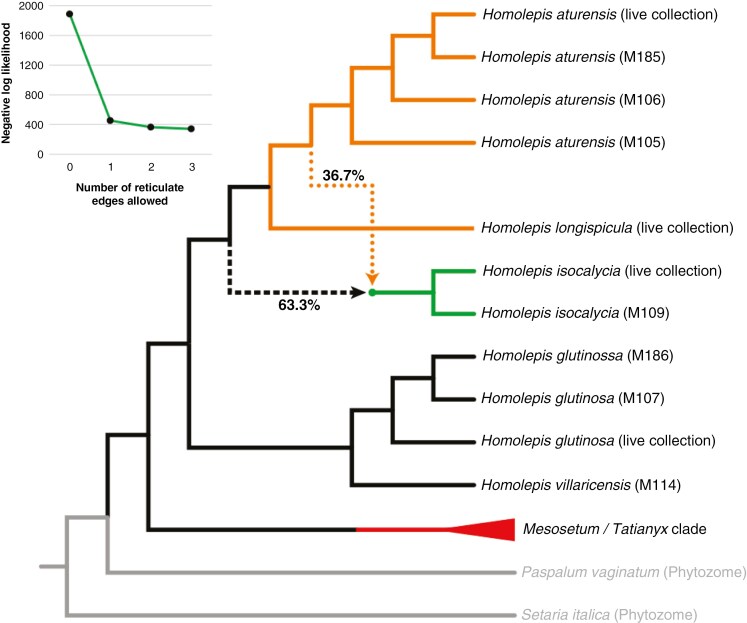
A phylogeny of *Homolepis* based on a concatenated super-matrix of 2858 genes and coalescent analyses of 4189 gene trees. Major and minor hybrid genetic contributions are denoted with dashed and dotted lines, respectively, and percentage values indicate the relative contribution of each branch. All nodes had full bootstrap and local posterior probability support from the super-matrix and coalescent analyses, respectively. Both methods placed *H. isocalycia* in a position consistent with the major (dashed) hybrid edge. Phylogenetic network analyses strongly supported a single hybridization event, with a major improvement in log-likelihood observed when one reticulation was allowed and no further improvement as the number of reticulations was increased (inset line graph). Clade colours represent C_3_ (black), sub-C_2_ (green), C_2_ (orange) and C_4_ (red) photosynthetic pathway use. Outgroups are coloured grey.

**Fig. 10.**
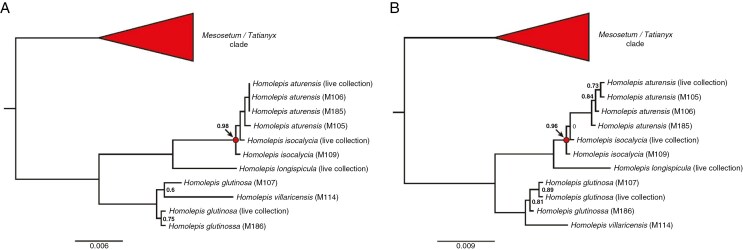
Gene trees for *Homolepis* orthologues of (A) GLDP, locus Pavag08G173400 in *Paspalum vaginatum*) and (B) one of two copies of the L-subunit of GDC (locus Pavag03G142400 in *P. vaginatum*).The red circles on each tree denote the focal node where *H. isocalycia* branches. Node support values were calculated with the Shimodaira–Hasegawa test implemented in FastTree, and values >0.95 are omitted, except for the red focal node. Branch lengths and scale bars denote substitution rates per site.

Phylogenetic inference based on a concatenated super-matrix of 2858 loci and coalescent-based analysis of 4189 gene trees (Supplementary Data Fig. S7) yielded the same tree topology with full support (100 % bootstrap support or 1.0 local posterior probability) at all nodes. The resulting combined phylogenetic tree shows two monophyletic clades without introgression within *Homolepis*, comprising *H. glutinosa* and *H. villaricensis*, and the other containing the reduced *Γ* species *H. isocalycia*, *H. longispicula* and *H. aturensis* (Fig. 9). Allowing a single reticulation within the tree via coalescent-based phylogenetic network inference improved the likelihood of the tree, with a 76 % improvement in the negative log-likelihood score (1888 to 453, lower being better) over a topology having no reticulation (Fig. 9, inset). Increasing the number of allowed reticulations beyond one resulted in no further improvement, allowing us to conclude that a single hybridization event likely occurred in the evolutionary history of *Homolepis*. A single reticulation supports a hypothesis that *H. isocalycia* is of hybrid origin, with a 36.7 % genetic contribution from *H. aturensis* along a reticulate edge in the tree. To further evaluate this hypothesis, we generated trees for five genes encoding the P, T, L, and H subunits of glycine decarboxylase. The gene encoding the P-subunit and one of two conserved copies of the gene coding for the L-subunit in *H. isocalycia* showed evidence of introgression from *H. aturensis* (Fig. 10). Specifically, the *H. isocalycia* branches for the P- and L-subunits of glycine decarboxylase were nested within a clade formed by *H. longispicula* and *H. aturensis*. These genes thus exhibit evolutionary histories consistent with *H. isocalycia* having inherited a potentially C_2_-adapted isoform from *H. aturensis* along the minor hybrid edge depicted in Fig. 8.

## DISCUSSION

The eudicot model of C_4_ evolution postulates the evolutionary transition from the C_3_ to the C_2_ character state begins with photosynthetic activation of the BS cells through the enhancement of organelle numbers in BS tissue, and then proceeds through a gradual upregulation of the C_2_ pathway, after which the C_4_ pathway is engaged and optimized (Fig. 1; Sage *et al*., 2012; Leung *et al*., 2024). Using the structural and functional character states described in Fig. 1 as a guide, we conclude that *H. glutinosa* is a C_3_ species. Gas exchange responses demonstrate that it has a high *Γ* while its BS cells lack organelle enrichment. Its sister species, *H. villaricensis*, also appears to be C_3_ based on no obvious organelle mass in the BS cells of rehydrated herbarium specimens and a C_3_-like δ^13^C value. In *H. isocalycia*, a *Γ* of 44 µmol mol^−1^ and its BS ultrastructure, to include centripetal positioning of mitochondria, are between values for the C_3_ and C_2_ species. Importantly, although GDC is enhanced in BS cells, a substantial fraction of GDC is also present in the M tissue of *H*. *isocalycia*. As a result of these physiological and cellular parameters, *H. isocalycia* clustered near the C_2_ species in a principal component analysis (Supplementary Data Fig. S6). We therefore conclude that *H. isocalycia* is a sub-C_2_ species as the evidence indicates it is operating a C_2_ cycle but lacks the near-total restriction of GDC to the BS cells that is typical of fully C_2_ species. *Homolepis isocalycia* represents the fifth sub-C_2_ species identified, after *Neurachne lanigera*, *Flaveria sonorensis*, *F. angustifolia* and *Tribulus astrocarpus* (Khoshravesh *et al*., 2020*b*; Adachi *et al*., 2023; Leung *et al*., 2024). However, while its physiology and biochemistry indicate that *H. isocalycia* is sub-C_2_, the question of whether it is representative of a *bona fide* evolutionary intermediate is uncertain, given our phylogenetic assessment that it arose via hybridization between *H. aturensis*-like C_2_ ancestors and a putative C_3_-like ancestor rooted near the base of the *Homolepis* phylogeny.

*Homolepis longispicula* and *H. aturensis* are well-developed C_2_ species, as indicated by *Γ* values between 15 and 21 µmol mol^−1^, as well as enhanced percentages of chloroplast, peroxisome and mitochondria area in the BS tissue relative to C_3_  *Homolepis* species. In addition, *H. longispicula* and *H. aturensis* position all BS mitochondria in a centripetal position, and >86 and 91 % of leaf GDC is allocated to the BS tissue of *H*. *longispicula* and *H*. *aturensis*, respectively (Figs 5 and 6; Table 5). Our transcript expression and enzyme activity analyses show no evidence for nascent activity of C_4_ cycle enzymes in *H. aturensis* or *H. isocalycia*, demonstrating they are not of the C_2_+ phenotype (Supplementary Data Tables S2 and S3). If the sister position of *Homolepis* to the C_4_  *Mesosetum* clade reflects phenotypes present in their common ancestors (as suggested in Grass Phylogeny Working Group II, 2012), then our results support the C_2_ condition being a ‘bridge’ for the evolution of C_4_ photosynthesis in the *Mesosetum* clade of the Arthropogoninae. Notably, the spatial organelle and GLDP immunolabelling patterns in *Homolepis* are similar to those observed in the C_2_ grasses *Steinchisma* (Otachyrinae; Brown *et al*., 1983; Khoshravesh *et al*., 2016), *Neurachne* (Neurachninae; Hattersley *et al*., 1986; Khoshravesh *et al*., 2020*b*) and eudicots in the genera *Alternanthera*, *Chenopodium*, *Euploca*, *Euphorbia*, *Flaveria*, *Mollugo* and *Tribulus* (Muhaidat *et al*., 2011; Sage *et al*., 2011, 2014; Yorimitsu *et al.*, 2019; Adachi *et al*., 2023; Leung *et al*., 2024) and the Amaranthaceae tribe Salsoleae (Voznesenskaya *et al.*, 2013). These similarities support a common model of C_2_ evolution in eudicots and grasses as summarized in Fig. 1, rather than a divergent eudicot and grass path to C_2_ and C_4_ photosynthesis as indicated by the Bayesian analysis of Williams *et al.* (2013).

The δ^13^C values of −13.5 ‰ and the gas exchange characteristic (steep initial slope of the *A*/*C*_*i*_ response, the *Γ* near 0 µmol mol^−1^, a *A*_400_/*A*_sat_ value near 1 and a *C*_i_/*C*_a_ ratio that is half that of the C_3_ species) demonstrate that *M. loliiforme* is a well-developed C_4_ species and not a sub-C_4_ species. Transcript expression and structural data support a conclusion that *M. loliiforme* is an NADP-ME C_4_ photosynthetic subtype as suggested by Brown (1977), Hattersley and Watson (1992) and Giussani *et al.* (2001). For example, the BS chloroplasts in *M. loliiforme* have reduced grana typical of BS chloroplasts in NADP-ME plants (Dengler and Nelson, 1999). Anatomical criteria associated with each C_4_ photosynthetic subtype in grasses include the number of sheath layers surrounding the xylem and phloem [outer parenchymatous sheath (BS) and inner sheath (mestome sheath)], continuity of the mestome sheath, and which vascular sheath layer is co-opted as the site of C_4_-acid decarboxylation (Hattersley and Watson, 1975; Edwards and Voznesenskaya, 2011). We identify two sheath layers surrounding the xylem and phloem of the primary and secondary veins in *M. loliiforme* leaves, while a single sheath layer is reported by Brown (1977) (*M*. *loliiforme*, *M*. *filifolium*), Hattersley and Watson (1992) (*M*. *pittieri*) and Giussani *et al.* (2001) (*M*. *chaseae*). We demonstrate that the outer, organelle-rich parenchymatous sheath (BS) in *Homolepis* is where photorespired CO_2_ is trapped and refixed, while in *M*. *loliiforme* this outer sheath (BS) is the site of C_4_-acid decarboxylation and CO_2_ concentration. In *M. loliiforme* the outer sheath is filled with enlarged, centrifugally placed chloroplasts, which supports the conclusion that it is the Kranz sheath layer where CO_2_ is concentrated during C_4_ photosynthesis. The anatomical characteristics of *M. loliiforme* lead us to classify its Kranz anatomy as the eriachneoid type, which occurs in species of the *Eriachne* clade of C_4_ photosynthesis in the grass subfamily Micrairoideae (Edwards and Voznesenskaya, 2011). *Mesosetum* represents a second, independently evolved case of this form of Kranz anatomy. This type of Kranz anatomy is distinct from a commonly observed classical NADP-ME type of anatomy, where the mestome is absent and CO_2_ concentration occurs in an outer parenchymatous sheath with centripetal chloroplasts (Edwards and Voznesenskaya, 2011). Other NADP-ME grass Kranz anatomy forms include those with either the inner sheath (aristidoid, neurachneoid) or outer sheath (stipagrostoid) forming the Kranz layer, where CO_2_ is concentrated. In contrast to *M. loliiforme*, chloroplasts with reduced grana are centripetally positioned in stipagrostoid types (Edwards and Voznesenskaya, 2011). The range of anatomical types in NADP-ME grasses demonstrate there is no linkage between biochemical subtype and the sheath layer co-opted for C_4_ photosynthesis, such that it becomes necessary to characterize the biochemistry and anatomy of each distinct C_4_ lineage to understand how the C_4_ pathway was evolutionarily assembled in the grasses. One challenge remaining is to fully characterize the anatomical type throughout the Arthropogoninae, to understand the degree to which a Kranz form can vary, and to examine whether related yet distinct C_4_ clades express similar forms of Kranz anatomy. Brown (1977), Giussani *et al.* (2001) and Hattersley and Watson (1992) note the members of the C_4_ clade containing *Arthropogon*, *Mesosetum* and *Tatianyx* have variable sheath numbers, suggesting either switching between Kranz type or independent evolution of Kranz anatomy in what appears to be a monophyletic C_4_ clade.

In *Mesosetum*, certain cellular patterns stand out when considering possible evolutionary pathways to C_4_ from putative *Homolepis*-like ancestors. First, the BS chloroplast numbers are reduced in *M. loliiforme* yet the chloroplasts are substantially enlarged relative to C_2_  *Homolepis* species. A similar pattern occurs in *Heliotropium* and *Flaveria*, where the C_2_ species and sub-C_4_ species (*F*. *brownii*) have many smaller chloroplasts in comparison with the C_4_ species, which have fewer, larger chloroplasts (Ku *et al.*, 1983; Holaday *et al*., 1984; McKown and Dengler, 2007; Muhaidat *et al*., 2011). These similarities highlight how BS chloroplast numbers decrease and chloroplast size often expands during the optimization phase of C_4_ evolution (Sage *et al*., 2012). Second, BS chloroplasts are positioned centrifugally adjacent to a thickened cell wall with a suberin lamella in *M. loliiforme* as well as *M*. *ferrugineum*, as they are in many but not all C_4_ grass lineages (Supplementary Data Fig. S5; Hattersley and Browning, 1981; Eastman *et al*., 1988; Dengler and Nelson, 1999). In grasses, the suberin lamella is proposed to enhance resistance to diffusive CO_2_ efflux following decarboxylation of C_4_ acids (Hattersley *et al*., 1986; von Caemmerer and Furbank, 2003; Danila *et al*., 2021). This centrifugal positioning of fewer, enlarged chloroplasts adjacent to a thickened, suberized wall stands in contrast to the centripetal positioning of many small chloroplasts in thin-walled BS cells in the C_2_  *Homolepis* species. In the C_2_  *Homolepis* species, the width of the BS cells may be more important for creating a diffusive barrier preventing rapid efflux of CO_2_ from centripetally positioned mitochondria, particularly since the outer BS wall appears to lack a suberin layer. Assuming the C_2_  *Homolepis* species represent evolutionarily intermediate character states that were present in the ancestors of the C_4_  *Mesosetum* clade, the results here indicate that a shift in BS chloroplast size/number and positioning occurred within the BS with the development of a thickened, suberized cell wall during the C_2_ to C_4_ transition. If this hypothesis is correct, then this would represent the first evidence for such a repositioning of chloroplasts from C_3_–C_4_ intermediate to C_4_ in parenchymatous BS cells within an evolutionary clade. Such a switch would imply a marked reorganization of cytoskeletal control over organelle positioning (Kong *et al*., 2024) during the later stages of C_4_ evolution. The pattern of chloroplast centrifugal positioning in *Mesosetum* differs from all C_4_ eudicot clades, in which enlarged chloroplasts remain in a centripetal position within a BS cell which also lacks suberin lamella (Dengler and Nelson, 1999). In *Neurachne* and *Alloteropsis*, the only grass lineages where C_2_ species are known to be immediate sisters to C_4_ clades, the inner, mestome sheath is co-opted to serve as the site of CO_2_ concentration in Kranz anatomy. There is no major repositioning of chloroplasts during the C_2_ to C_4_ transition in *Alloteropsis* and one of the two C_4_  *Neurachne* species, *N*. *munroi*. (Lundgren *et al.*, 2019; Khoshravesh *et al.*, 2020b).

A notable observation within the *M. loliiforme* BS is a reduction in mitochondria coverage to C_3_-like values while chloroplast coverage of the BS cell area increases 8-fold over the C_3_ values observed in *H. glutinosa*. This contrasting pattern of chloroplast versus mitochondria coverage also supports classification of *M. loliiforme* as an NADP-ME subtype of C_4_ photosynthesis, which has fewer BS mitochondria than NAD-ME malic enzyme species (Hatch *et al*., 1975; Khoshravesh *et al*., 2020*a*). In NAD-ME subtypes of C_4_ photosynthesis, the decarboxylating enzyme NAD-ME is mitochondrial, leading to high demand for BS mitochondria (Hatch, 1987). NADP-ME is chloroplastic, which likely contributes to the expansion of chloroplast size observed in the BS of *M. loliiforme*. Also, we observed a large drop in GLDP immunogold particle density in the BS of *M. loliiforme*, reflecting decreased metabolic demand for glycine decarboxylation brought about by low photorespiration rates in this C_4_ plant.

The evolvability of C_4_ photosynthesis in grasses is posited to increase when the proportion of BS is higher than 15 % of planar leaf area when viewed in cross-section (Christin *et al*., 2013). The proportion of BS in the C_3_  *H. glutinosa* is 27 % of leaf photosynthetic tissue, which is consistent with the hypothesis of Christin *et al*. (2013). In addition, we observed that *H. glutinosa* positioned two-thirds of its BS mitochondria in the inner BS against the vascular tissues (Fig. 1). Numerous C_3_ grasses commonly position about two-thirds of their BS mitochondria centripetally, which could help recycle photorespiratory CO_2_ in leaves (Hatakeyama and Ueno, 2016; Khoshravesh *et al*., 2016); however, many of these C_3_ grasses do not exceed the 15 % proportion of planar leaf area invested in the BS (Christin *et al*., 2013). The centripetal position of most mitochondria in the BS of C_3_ grasses combined with an enhanced percentage of BS tissue could enable some evolutionary lines to boost the fraction of BS mitochondria in a centripetal position, eventually to >90 %. Moving nearly all mitochondria into a centripetal position is a key trait that delineates the proto-Kranz character state and is hypothesized to be an early phase of C_2_ and C_4_ evolution (Muhaidat *et al*., 2011; Sage *et al.*, 2013). If so, the high fraction of centripetal mitochondria in *H. glutinosa* and other C_3_ grasses in combination with a proportion of BS higher than 15 % of planar leaf area could be a key enabler for the initiation of C_4_ evolution and could help explain why there are so many C_4_ origins in the grass family (>20; Sage, 2016).

Surprisingly, the BS cell width, length and volume decrease as *Γ* declines in *Homolepis* and *Mesosetum*, such that the volume of an individual BS cell in the C_4_  *Mesosetum* plants is just 7 % of that of the BS cells in the C_3_  *H. glutinosa*. Over half of the size reduction in the BS cells of *M. loliiforme* relative to *H. glutinosa* was due to decreasing cell length. This decline in BS length with decreasing *Γ* appears to begin during the transition from C_3_ to C_2_ phenotypes, as indicated by the statistically lower BS length observed in the sub-C_2_  *H. isocalycia* (Table 3). In C_2_ and C_4_  *Neurachne* species, the mestome sheath cells (where CO_2_ is concentrated) also decline in length during C_2_ evolution (Khoshravesh *et al*., 2020*b*). These shifts in 3-D features of the carbon-concentrating cells in the C_2_ species of *Homolepis* and *Neurachne* are likely caused by an increase in cell division along the base-to-tip (lengthwise) axis of the leaf, regardless of whether the carbon-concentrating cell is an outer BS sheath (as in *Homolepis*) or an inner mestome sheath (as in *Neurachne*). The functional significance of this change has been hypothesized to result in shorter, wider cells that could allow more organelles per length of sheath tissue and/or greater resistance to outward diffusion of CO_2_ (Khoshravesh *et al*., 2020*b*). In *Homolepis* and *Mesosetum,* however, the increase in BS cell number along the length of the BS tissue is associated with narrower BS cells as *Γ* declines, and greater organelle content within the BS. We hypothesize that the acquisition of a suberized BS wall by the C_4_  *Mesosetum* clade may allow sufficient resistance regardless of BS width, while the increase in organelle content reflects regulatory control of organelle size and numbers that is distinct from that controlling BS cell size. For example, greater activity of transcription factors such as *GLK* could boost organelle content without changing BS cell size, as observed in rice (Wang *et al*., 2017).

Peroxisome patterns are infrequently examined in C_2_ species. Here we observed an increase in the allocation of leaf peroxisomes to the BS tissue of the C_2_ relative to the C_3_ species, demonstrating that the partitioning of the photorespiratory pathway to the BS involves more than just the shift in GDC activity. Almost a third of the leaf peroxisome area is present in the BS of *H. aturensis* and *H. longispicula*, compared with <2 % in the BS of the C_3_  *H. glutinosa*. Assuming functionality tracks the relative planar area of peroxisomes observed in the BS, this would indicate a substantial fraction of the photorespiratory flux from the BS to the M tissue occurs as glycerate, rather than serine as is often portrayed (Bauwe, 2011; Mallman *et al*., 2014; Mercado and Studer, 2022). If the principal flux into the BS remains glycine and the flux from BS to the M tissue is glycerate, there would be significant implications for nitrogen (N) balancing that could require upregulation of C_4_ pathway components (Mallmann *et al*., 2014; Walsh *et al*., 2023). For example, if glycine is produced in the M peroxisome, while glycerate is produced in the BS peroxisome, then elements of the C_4_ pathway could be needed to provide carbon skeletons to transaminate N from serine to form glutamate in the BS peroxisomes, and then to transport this glutamate to the M peroxisomes to enable glycine formation from newly formed glyoxylate (Mallmann *et al*., 2014). Alternatively, a significant fraction of the photorespiratory cycle, notably the cycling of amino-N within peroxisomes, may be localized to the BS, with glycolate entering the BS rather than glycine, while glycerate exits the BS (Borghi *et al*., 2022). Localizing the photorespiratory N cycle within the BS may reduce potential problems of N imbalance between M and BS cells (Mallmann *et al*., 2014), and thus reduce a need to upregulate elements of the C_4_ cycle. We hypothesize that C_2_ lineages lacking close C_4_ relatives (for example *Steinchisma*) may have substantial enhancement of peroxisome numbers and/or size in the BS tissue, which could reduce evolutionary pressure to upregulate C_4_ cycle enzymes in support of C_2_ metabolism. Consistently, Khoshravesh *et al*. (2016) document that BS cells of *Steinchisma hians* have, on average, more peroxisomes in BS cells relative to the C_3_ proto-Kranz *S. laxum*.

### Phylogenetic pattern and significance of C_3_ × C_2_ hybridization

Both the concatenated super-matrix tree and a coalescent tree placed *Homolepis* as sister to the C_4_ Arthropogoninae clade as represented here by *M. loliiforme* (Fig. 8; Supplementary Data Fig. S7) (see also Arthropogoninae phylogenies by Morrone *et al*., 2007; Grass Phylogeny Working Group II, 2012; Zuloaga *et al*., 2018; Acosta *et al*., 2019; Gallaher *et al*., 2022). Within *Homolepis*, two clades are evident, one with the C_3_ species *H. glutinosa* and probable C_3_ species *H. villaricensis*, and a second with the sub-C_2_  *H. isocalycia* plus the C_2_ species *H. longispicula* and *H. aturensis*. In the Arthropogoninae phylogeny of Gallaher *et al.* (2022), *Homolepis* branches immediately sister to a C_4_ clade with 37 species in the genera *Achleana*, *Altoparadisium*, *Arthropogon*, *Keratochlaena*, *Mesosetum*, and *Tatianyx* (taxonomy follows Kellogg, 2015). These genera plus *Homolepis* in turn arise from a node that branches sister to a C_3_ clade (*Stephostachys*, one species), and a distinct ‘*Apochloa*’ clade comprising four C_3_ genera (*Apochloa*, *Oplismenopsis*, *Phanopyrum* and *Triscenia*; 18 species in total) and four C_4_ genera (*Canastra*, *Coleataenia*, *Cyphonanthus* and *Oncorachis*; 14 species in total). The sister position of the *Apochloa* clade to the *Homolepis*/*Mesosetum* C_4_ clade suggests two or three independent origins of C_4_ photosynthesis in the Arthropogoninae, making it a prolific clade for C_4_ evolution (Gallaher *et al*., 2022). Within the *Homolepis*/*Mesosetum* clade, the sister position of *Homolepis* to the C_4_ lineages supports a hypothesis that its C_3_–C_4_ intermediate species reflect ancestral intermediate states for *Mesosetum* and related genera. However, a ladder-like phylogenetic progression from C_3_ through various intermediate states to C_4_ is not apparent in the Arthropogonineae phylogeny as it is in the *Flaveria* phylogeny (Adachi *et al*., 2023). Instead, the phylogenetic pattern is suggestive of intermediate ancestral states being present in the phylogenetic backbone below the *Homolepis* and C_4_  *Mesosetum* clades. This pattern is also evident in other C_3_ to C_4_ clades, notably *Alternanthera*, *Euploca* and *Neurachne* (Sanchez del Pino *et al*., 2012; Khoshravesh *et al*., 2020*b*; Frohlich *et al*., 2022).

Hybridization between non-C_4_ and C_4_ species has been invoked as both a driver of C_4_ evolution by introgression of C_4_ genes into ancestral, non-C_4_ populations (Christin *et al*., 2012*a*; Dunning *et al*., 2017; Pereira *et al*., 2023), and alternatively as a source of apparent intermediate phenotypes that may not reflect *bona fide* states of evolutionary intermediacy and thus could mislead evolutionary models (Kadereit *et al*., 2017; Tefarikis *et al*., 2022). In *Homolepis*, our phylogenetic analyses placed *H. isocalycia* in a position consistent with a hybrid origin, with gene flow evident between *H. aturensis* and an ancestor of *H. longispicula*. Further support for hybridization in the ancestry of *H. isocalycia* comes from its chromosome count (40) compared with *H. aturensis* (20–22), consistent with an allopolyploidy event (Gould and Soderstrom, 1967, 1970; Pohl and Davidse, 1971). Our phylogenetic network analyses strongly supported a single hybridization event, with a major improvement in log-likelihood being observed when one reticulation was allowed, but no further improvement as the number of reticulations was increased. Furthermore, the genes encoding the GDC subunits exhibited evolutionary histories consistent with *H. isocalycia* having inherited potentially C_2_-adapted P and L subunits from an *H. aturensis* ancestor along the minor hybrid edge illustrated in Fig. 8. These results demonstrate that hybridization can generate variation within the C_3_–C_4_ intermediate character state, with the sub-C_2_ phenotype being a possible result. No evidence exists for a C_3_ × C_4_ hybrid in *Homolepis*, leading us to conclude the C_2_ state evolved *de novo*, as is hypothesized in numerous C_3_ to C_4_ transitions (Christin *et al*., 2010; Lundgren *et al*., 2016; Frohlich *et al*., 2022; Adachi *et al.*, 2023). Whether the sub-C_2_ phenotype represents a *bona fide* evolutionary intermediate in *Homolepis* cannot be concluded from the results of the present study, but is supported by phylogenetic patterns in *Flaveria* and *Neurachne* (Khoshravesh *et al*., 2020*b*; Adachi *et al*., 2023).

## Conclusions

The evolution of C_4_ photosynthesis consists of two phases, one being the rise of the C_2_ character state and the second being the transition from C_2_ to C_4_. In the present study, we provide a detailed physiological and anatomical assessment of one *Mesosetum* and four *Homolepis* species to improve understanding of C_2_ and C_4_ evolution in the grasses and how it compares with patterns of C_2_ and C_4_ evolution in eudicots. We conclude that many physiological and structural features contributing to photosynthetic activation of the BS and C_2_ evolution within *Homolepis* are similar to those in eudicots, in contrast to predictions of dissimilarity by a Bayesian hierarchy model (Williams *et al*., 2013). Increases in the BS of centripetally located organelles, GLDP and cell division in the basal–apical direction of the leaf are associated with BS activation in C_2_  *Homolepis* species, indicating that substantial modification of the BS occurs in the earliest phases of C_4_ evolution, specifically, during the sub-C_2_ phase. Developmental comparisons of leaf growth between *Homolepis* and *M. loliiforme* could therefore be valuable for establishing the mechanism controlling leaf modification during Kranz anatomy evolution in grasses. We also show that hybridization can produce intermediate phenotypes between C_3_ and C_2_ lineages in *Homolepis*, demonstrating the importance of considering hybridization when addressing questions of C_2_ and C_4_ evolution (Kadereit *et al*., 2017; Tefarikis *et al*., 2022). Using data compiled in the past decade, it should now be possible to theoretically explore similarities and differences between the evolution of C_2_ photosynthesis in monocots and eudicots in a manner that was not possible with the more limited data set available to Williams *et al.* (2013). In addition to using the Williams *et al*. model to incorporate more recent data sets, the evolutionary landscape model of Heckmann *et al.* (2013), which predicted the rise of the C_4_ pathway from the C_2_ condition, could be extended to address origins of the C_2_ pathway using the much richer data set available today.

## SUPPLEMENTARY DATA

Supplementary data are available at *Annals of Botany* online and consist of the following. Table S1: δ^13^C raw data and herbarium vouchers. Table S2: transcript values for C_4_ cycle enzymes. Table S3: enzyme activity of four C_4_ cycle enzymes. Appendix S1: detailed phylogenomics methodology. Figure S1: leaf anatomy of rehydrated *H. villaricensis* from herbarium specimens. Figure S2: TEM micrographs of BS cell ultrastructure and GLDP labelling patterns in four *Homolepis* species. Figure S3: TEM micrographs of mesophyll cell ultrastructure and GLDP labelling in four *Homolepis* species. Figure S4: TEM images of bundle sheath and mesophyll and immunogold labelling in *M. loliiforme*. Figure S5: TEM images of junction between M and BS cell walls illustrating absence (*Homolepis* species) or presence (*Mesosetum* species) of suberin lamella in cell wall. Figure S6: principal component analysis and component loadings. Figure S7: concatenated and coalescence-based phylogenetic trees used to generate Fig. 8.

mcae214_suppl_Supplementary_Tables

mcae214_suppl_Supplementary_Figures
