# Supplementary material for: Evolutionary diversification of C2 photosynthesis in the grass genus Homolepis (Arthropogoninae)
Source: Ann Bot. 2024 Dec 17;135(4):769–88. doi: 10.1093/aob/mcae214 (PMC11904902; doi:10.1093/aob/mcae214)
Supplement: mcae214_suppl_Supplementary_Tables [file mcae214_suppl_supplementary_tables.pdf]

SUPPLEMENTARY TABLES AND PHYLOGENOMICS METHODS

Pages 2-6. Supplementary Table S1: $\delta^{13}C$ raw data and herbarium vouchers

Page 7. Supplementary Table S2: transcript values for C_4 cycle enzymes

Page 8. Supplementary Table S3: enzyme activity of four C_4 cycle enzymes

Page 9-11. Appendix S1: detailed phylogenomics methodology

Supplementary Table S1: $\delta^{13}C$ values of live and herbarium specimens of species in the Arthropogineae examined in this study. Specimens used for DNA analysis are underlined and highlighted in bold.

Species	Collector	Location	Date	Herbarium	$\delta^{13}C$ value, ‰
Arthropogon					
A. filifolius	Filgueiras & Mendoca 1184	Federal District, Brazil	25 September 1985	NYBG	-12.2
A. filifolius	Filgueiras & Mendoca 1222	IBGE Ecology Reserve, Brazil	30 September 1986	NYBG	-12.8
A. filifolius	Filgueiras 1086	IBGE Ecology Reserve, Brazil	October 7, 1983	NYBG	-12.7
A. villosus	Mendoca & Alvarenga 629	IBGE Ecology Reserve, Brazil	22 May 1986	NYBG	-12.3
A. villosus	Irwin et al. 8309	Federal District, Brazil	14 September 1965	NYBG	-12.3
A. villosus	Kral et al. 75720	Goais, Brazil	29 November 1988	NYBG	-13.3
A. xerachne	Eiten & Sendulsky	Sao Paulo State, Brazil	31 May 1961	NYBG	-11.3
Homolepis					
H. aturensis	Breedlove 46870	Chiapas, Mexico	25 Oct 1980	NYBG	-28.2
H. aturensis	Aguilov 13031	Punta Renas Osa, Costa Rica	11 Dec 1980	NYBG	-29.6
H. aturensis	Smith et al. 1631	Madre De Dios, Peru	27 January 1989	NYBG	-31.7
H. aturensis	Solomon 7955	Beni, Bolivia	6 June 1982	NYBG	-31.1
H. aturensis	Nee 42378	Amazonia, Brazil	29 January 1992	NYBG	-31.3
H. aturensis	D. Atha 1238a	Toledo District, Belize	2 August 1995	NYBG	-29.8
H. glutinosa	Pohl 14126	Cartago, Costa Rico	4 August 1982	NYBG	-30.1

H. glutinosa	Pohl and Pinette 13253		23 June 1976	NYBG	-31.5
H. glutinosa	Koch and Fryxell 7724		6 March 1977	NYBG	-25.3
H. glutinosa	Atha et al. 6711	Puerto Rico	22 August 2008	NYBG	-29.8
H. glutinosa	Dory et al. 8266	Venezuala	27 Oct 1998	NYBG	-33.5
H. glutinosa	Boom & Gopaul 7291	Mazaruni River area, Guyana	14 April 1987	NYBG	-27.0
H. isocalycia	Godfry 2047		3 Nov 1965	NYBG	-27.8
H. isocalycia	Callejos et al. 3535	Colombia	15 May 1987	NYBG	-31.5
H. isocalycia	Pipoly 8650	Guyana, Mabura Hill	18 September 1986	NYBG	-29.3
H. isocalycia	Henkal and Williams 1994	Guyama, Demerara-Berbice	29 April 1993	NYBG	-27.4
H. isocalycia	Oldenburger et al. ON355	Suriname, Gebroader Mountains	26 October 1968	NYBG	-28.0
H. longispiculata	Ratter et al. 3356	Federal District, Brazil	3 August 1976	NYBG	-27.9
H. longispiculata	Irwin et al. SN	Federal District, Brazil	23 September 1965	NYBG	-27.2
H. longispiculata	Renvoize 973	Curitiba, Brazil	No date	NYBG	-27.2
H. longispiculata	Eiten & Eiten 6754	Minas Gerais, Brazil	21 November 1965	NYBG	-27.6
H. villaricensis	Morone 779	Missiones, Argentina	14 February 1996	NYBG	-33.4
H. villaricensis	Montes 27562	Missiones, Argentina	20 March 1958	NYBG	-32.0
H. villaricensis	Eichentes 747	Argentina	18 April 1945	NYBG	-30.0
H. villaricensis	Zuloaga et al. 5664	Argentina	20 April 1996	NYBG	-32.2
Mesosetum					
M. alatum	Irwin et al. 1998	Mato Grosso, Brazil	2 June 1966	NYBG	-12.0

M. annuum	Irwin et al. 21697	Northern Goias, Brazil	23 March 1968	NYBG	-10.8
M. arenarium	Irwin et al. 13337	Goias, Brazil	3 March 1966	NYBG	-11.1
M. arenarium	Filgueras & Burman 1106	Minas Gerais, Brazil	19 January 1984	NYBG	-11.8
M. blakei	Gentle 8299	Susan Creek, Belize	9 August 1954	NYBG	-12.1
M. cayense	Strudwick et al. 4381	Para, Brazil	8 August 1981	NYBG	-11.9
M. cayense	Plowman et al. 8696	Para, Brazil	11 February 1980	NYBG	-12.6
M. cayense	Anderson 7135	Goias, Brazil	14 March 1973	NYBG	-12.2
M. chasae	Coradin and Cordeiro 871	Brazil	26 October 1977	NYBG	-11.7
M. chasae	Petersen et al. 7564	Guyana	21 June 1989	NYBG	-12.6
M. chlorostachyum	Davidse et al. 16863	Venezuela	29 April 1979	NYBG	-13.6
M. chlorostachyum	Elisa de Paula et al, 10421	Brazil	March 18, 1909	NYBG	-11.0
M. comatum	Morone et al. 1000	Argentina	February 20, 1996	NYBG	-13.6
M. comatum	Zuloaga et al. 5329	Argentina	March 6, 1995	NYBG	-13.2
M. elytrochaetum	Irwin et al. 1270	Brazil	February 12, 1966	NYBG	-11.8
M. elytrochaetum	Irwin et al. 32768	Brazil	March 20, 1971	NYBG	-11.5
M. exacretum	Sendulsky 443	Brazil	December 9, 1971	NYBG	-11.0
M. ferrugineum	Ganev 2684	Brazil	December 21, 1973	NYBG	-12.0
M. ferrugineum	Harley et al. H50756	Brazil	January 10, 1992	NYBG	-11.8
M. ferrugineum	Irwin et al. 0660	Brazil	November 25, 1965	NYBG	-11.2
M. filifolium	Neil 4417	Zelaga, Nicaragua	13 June 1978	NYBG	-11.1

M. filifolium	Atha 1218	Belize	1 August 1995	NYBG	-12.2
M. filifolium	Davidse et al. 17417	Venezuela	May 8, 1979	NYBG	-12.4
M. loliiforme	Eckman 11350	Pinar del Rio Cuba	nd	NYBG	-11.1
M. gibbosum	Souza et al. 5238	Brazil	February 13, 1994	NYBG	-12.9
M. gibbosum	Pirani et al. H51473	Brazil	February 2, 1992	NYBG	-11.7
M. loliiforme	Eckman 1028	Pinar del Rio Cuba	25 July 1921	NYBG	-10.4
M. loliiforme	Sasaki and Sasaki 922	Brazil	13 January 2004	NYBG	-17.8
M. loliiforme	Irwin et al. 32477	Brazil	ND	NYBG	-13.3
M. loliiforme	Queiroz et al. 5214	Brazil	April 9, 1994	NYBG	-13.1
M. loliiforme	Davidse et al. 12108	Brazil	April 7, 1976	NYBG	-13.0
M. pappopharum	Luetzelberg 27072	Brazil	April 3, 1996	NYBG	-10.7
M. pappopharum	Swallen 4615	Brazil	May 16, 1934	NYBG	-10.8
M. pappopharum	Swallen 4453	Brazil	ND	NYBG	-11.2
M. pittieri	Allen 839	Nata region, Panama	12 September 1938	NYBG	-10.3
M. rottboelliodes	Huber and Tillett 5398	Venezuela	July 19, 1980	NYBG	-12.1
M. rottboelliodes	Huber 5259	Venezuela	June 18, 1980	NYBG	-13.8
M. rottboelliodes	Davidse 2737	Venezuela	October 31, 1971	NYBG	-12.2
M. rottboelliodes	Davidse and Huber 15015	Venezuela	April 13-15, 1978	NYBG	-13.5
M. wrightii	Eckman 990	Pinar del Rio, Cuba	10 October 1923	NYBG	-10.4
M. wrightii	Brother Leon 6430	Cuba	29 July 1916	NYBG	-9.9
Tatianyx					
T. arnacites	Zuloaga & Morrone 4691	Minas Gerais, Brazil	23 February 1993	NYBG	-11.3
T. arnacites	Queiroz et al. H51087	Bahia, Brazil	11 February 1992	NYBG	-12.9

T. arnacites	Carvalho et al. 6446	Mato Grosso, Brazil	27 December 1997	NYBG	-12.6

Supplementary Table S2: Expression values in transcript reads per million for genes encoding PEP carboxylase (PEPC), NADP-malic enzyme (NADP-ME), NAD-malic enzyme (NAD-ME) and pyruvate phosphate dikinase (PPDK). Expression values of these specific enzymes were obtained by mapping *Homolepis* and *Mesosetum* RNA-seq reads onto the *Paspalum vaginatum* genome (primary coding regions only) (https://phytozome-next.jgi.doe.gov). For each species, data for two plants is presented. Putative C_4 cycle isoforms were identified based on elevated expression in *Mesosetum* and are highlighted in bold font. Note: RNA-seq data are not available for *H. aturensis* and *H. longispicula*.

Gene	*Paspalum vaginatum* Locus ID	*Homolepis glutinosa* 1	*Homolepis. glutinosa* 2	*Homolepus isocalycia* 1	*Homolepis isocalycia* 2	*Mesosetum loliiforme* 1	*Mesosetum loliiforme* 2
**PEPC**	Pavag03G282000	4.4	6.8	2.9	2.4	0.8	1.1
**PEPC**	Pavag07G119500	35.6	26.2	42.0	37.0	2.4	2.5
**PEPC**	Pavag02G149000	2.5	2.9	15.1	13.4	55.6	64.1
**PEPC**	Pavag04G101300	44.5	46.0	58.9	46.8	**583.0**	**660.2**
**PEPC**	Pavag10G158300	43.5	20.4	71.3	53.8	**973.8**	**1044.8**
**PEPC**	Pavag10G158400	44.3	22.4	82.3	64.4	**1007.5**	**1070.2**
**NADPME**	Pavag03G272700	4.1	4.5	2.1	0.5	0.4	0.6
**NADPME**	Pavag09G074700	8.2	12.7	42.2	31.2	45.2	19.4
**NADPME**	Pavag03G260700	263.1	420.8	112.0	109.5	62.3	66.5
**NADPME**	PavagK315800	324.9	520.0	122.8	114.2	73.9	80.2
**NADPME**	Pavag03G034200	147.1	193.9	210.4	199.3	**787.9**	**813.2**
**NADME**	Pavag02G284900	36.9	44.4	36.2	35.2	12.5	22.0
**NADME**	Pavag01G184100	147.0	108.5	68.1	66.4	41.2	65.6
**PPDK**	Pavag01G314100	7.7	8.8	9.2	7.8	1.5	2.0
**PPDK**	Pavag09G139800	51.9	36.1	161.4	130.4	**6187.0**	**4741**

Supplementary Table S3: Activities of principle C_4 cycle enzymes in three *Homolepis* species, and for a C_4 comparison, *Gomphrena serrulata* (Amaranthaceae). At the time of assay, *Mesosetum loliforme* and *Homolepis longispicula* had died. Means ± SE, N=3 per species. Statistically distinct groups at p<0.05 by a one-Way ANOVA and Tukey's post hoc test are indicated with different letters following SE values. Data were log-transformed if normality failed for the test. * and ** indicate statistical groups at p<0.05 when on the three *Homolepis* species are compared.

Enzyme	*Homolepis glutinosa*	*Homolepis isocalycia*	*Homolepis aturensis*	*Gomphrena serrulata*
Enzyme	µmol mol^{-1} chlorophyll s^{-1}	µmol mol^{-1} chlorophyll s^{-1}	µmol mol^{-1} chlorophyll s^{-1}	µmol mol^{-1} chlorophyll s^{-1}
PEP carboxylase	10.9±1.2a	15.2±7.2a	18.4±1.8a	296.7±43.0b
NADP malic enzyme	15.4±2.8a	6.2±1.9a	6.5±1.4a	75.7±12b
NAD-malic enzyme	32.6±8.7a	69.2±22.7a	61.0±31.8a	24.8±9.8a
Malate dehydrogenase	2778±431a	1657±118a	2708±325a	983±456a

SupplementaryAppendix S1: Detailed methodology for generating the phylogenetic trees in Figures 8 and 9.

DNA preparation and sequencing

Dried leaves from herbarium specimens indicated in bold in Supplemental Table S1 were sampled for genomic DNA analysis by the Beijing Genomics Institute (BGI). Genomic DNA of living *Homolepis aturensis* was sequenced by the Centre for the Analysis of Genome Evolution and Function (CAGEF) at the University of Toronto (cagef.utoronto.ca).
The cetyltrimethylammonium bromide (CTAB) method was used to extract high-quality DNA from died leaves which was further used to construct DNA Nanoball (DNB) library for short read sequencing, and PacBio library for single-molecule real-time (SMRT) sequencing. For DNB-seq, the sequence was fragmented to 300-500 bp in size and amplified to nanoball which was then sequenced on DNB-seq platform with paired-end PE150 strategy. For *H. aturensis*, *g*enomic DNA was extracted using the CTAB method, sheared with Covaris, and used for MGI-PCR-free library prep. Sequencing was conducted with the MGI DNBSEQ-G400RS FCL platform generating 150bp paired-end reads. All other sequence data used in the tree was downloaded from the NCBI short-read archive.

Ortholog selection and reference-based assembly

A set of 9,279 genes which are conserved between Setaria italica and *Paspalum vaginatum* were identified from the version 2.2 and 3.1 genome annotations, respectively, downloaded from Phytozome (https://phytozome-next.jgi.doe.gov/). This set of genes were defined by the following BLAST criteria: gene pairs between the two species must be each other's reciprocal best hit with an e-value cut-off of 1e-50, and neither gene may have an endogenous paralog with a bitscore that is greater than 50% of the identity bitscore. The *Paspalum* genes from this set were used as a reference for mapping paired-end genomic DNA from *Homolepis*, *Mesosetum*, and *Tatianyx* samples in order to generate reference assemblies. This process consisted of the following steps: reads were mapped using Hisat2 (v2.2.1; Kim *et al.,* 2019) with parameters --score-min L,0.0,-0.875 --no-unal --no-discordant, with the results piped into the SamTools (v.1.18; Danecek *et al.*, 2021) "sort" command to generate sorted BAM files; VCF files were generated using the BCFTools (v.1.18; Danecek *et al.*, 2021) "mpileup" command; and reference-based assemblies were generated using a Python script. Variant frequency information contained in the VCF files determines whether the read mapping data exhibits evidence of multiple paralogous genes collapsing to a single reference as follows: if the second most frequent variant at a given site is at least 50% as frequent as the most frequent, that site is marked as heterozygous and the site called as a gap in the resulting assembly, and if more than 5% of sites are heterozygous in this manner the assembly is rejected. A minimum read depth of 3 was required, with all sites below this threshold being called as a gap. Insertions in the reference sequence are ignored and deletions are changed to gaps such that assembled sequences maintain alignment to the reference sequence. The reference-based alignments for each gene were then trimmed using trimAl (v1.4; Capella-Gutiérrez *et al.,* 2009) with the -gt 0.75 parameter to remove sites in the alignment comprised of 25% or more gaps. Next, sequences in the alignments which were comprised of 75% or more gaps were removed. A subset of 4,189 genes were selected for gene tree construction for coalescent-based phylogenetic methods based on retaining all taxa after gap removal and presence of at least 100 phylogenetically informative sites in the alignment (defined as sites with variants

present in two or more sequences). A smaller subset of 2,858 genes with a maximum alignment length of 2,000 bp was selected from this for concatenation-based phylogenetic inference in order to improve computation time.

Phylogenetic inference and analysis of hybridization

Phylogenetic inference on the concatenated super-matrix was conducted using RaxML-ng (v1.2.0; Kozlov *et al.*, 2019) with parameters --all for conducting best-tree search and bootstrap support analysis in one run and --bs-trees 100 for 100 bootstrap replicates. The GTR+G+I model was specified for each partition. Coalescent-based phylogenetic inference was conducted using ASTRAL (v5.7.1; Zhang *et al.*, 2018) on 4,189 gene trees which were generated using FastTree (v2.1.10; Price *et al.,* 2010) with parameters -nt -gtr -gamma. In addition to standard coalescent-based phylogenetic inference, the SNaQ function in the PhyloNetworks Julia package (Solís-Lemus *et al.*, 2017) was used to infer whether hybridization had occurred. SNaQ allows a maximum number of reticulate edges within a tree to be specified, and the appropriate number to allow is inferred based on a "slope heuristic" – that is, if allowing one more reticulation in the tree results in a substantial log-likelihood improvement, the reticulate edge is likely justified, but once allowing addition reticulation results in minimal improvement over-fitting is more likely (Solís-Lemus *et al.*, 2017). SNaQ was run repeatedly in parallel for 0, 1, 2, and 3 allowed reticulations, with a single run specified per command and a stopping criterion (the NFail argument, specifying the maximum number of times in a row that new topologies are proposed and rejected) of 100, which is more stringent than the default of 75. This increased stringency coupled with a minimum of 600 independent runs per allowed number of reticulations represents an exhaustive search of the phylogenetic network space to ensure the best network was found for each number of allowed reticulations. Because we found evidence of a hybrid origin for *H. isocalycia*, with its sub-C_2 phenotype possibly the result of gene flow from the C_2 species *H. aturensis*, gene trees were generated for five genes encoding the P, T, L, and H subunits of glycine decarboxylase (present as single genes for all but the L-subunit, where two conserved paralogs exist in Poaceae). The *Paspalum* genes encoding these subunits are Pavag08G173400 (P), Pavag08G054300 (H), Pavag03G142400 (L), Pavag03G143800 (L), and Pavag06G233900 (T). Reference-based assemblies and alignments for these genes were generated via the same process as the genes used in the species phylogeny, and gene trees were generated using FastTree in the same manner as those used for coalescent-based phylogenetic inference.

Literature Cited

Capella-Gutiérrez S, Silla-Martínez JM, Gabaldón T. **2009**. trimAl: a tool for automated alignment trimming in large-scale phylogenetic analyses. *Bioinformatics* **25**: 1972–1973.

Danecek P, Bonfield JK, Liddle J, Marshall J, Ohan V, Pollard MO, Whitwham A, Keane T, McCarthy SA, Davies RM, *et al.* 2021. Twelve years of SAMtools and BCFtools. *GigaScience* **10**: giab008.

Kim D, Paggi JM, Park C, Bennett C, Salzberg SL. **2019**. Graph-based genome alignment and genotyping with HISAT2 and HISAT-genotype. *Nature Biotechnology* **37**: 907–915.

Kozlov AM, Darriba D, Flouri T, Morel B, Stamatakis A. **2019**. RAxML-NG: a fast, scalable and user-friendly tool for maximum likelihood phylogenetic inference. *Bioinformatics* **35**: 4453–4455.

Price MN, Dehal PS, Arkin AP. **2010**. FastTree 2--approximately maximum-likelihood trees for large alignments. *PloS One* **5**: e9490.

Solís-Lemus C, Bastide P, Ané C. **2017**. PhyloNetworks: A Package for Phylogenetic Networks. *Molecular Biology and Evolution* **34**: 3292–3298.

Zhang C, Rabiee M, Sayyari E, Mirarab S. **2018**. ASTRAL-III: polynomial time species tree reconstruction from partially resolved gene trees. *BMC Bioinformatics* **19**: 153.
