# Supplementary material for: Evolutionary diversification of C2 photosynthesis in the grass genus Homolepis (Arthropogoninae)
Source: Ann Bot. 2024 Dec 17;135(4):769–88. doi: 10.1093/aob/mcae214 (PMC11904902; doi:10.1093/aob/mcae214)
Supplement: mcae214_suppl_Supplementary_Figures [file mcae214_suppl_supplementary_figures.pdf]

Pereira Alvarenga *et al*. Supplementary Figure S1

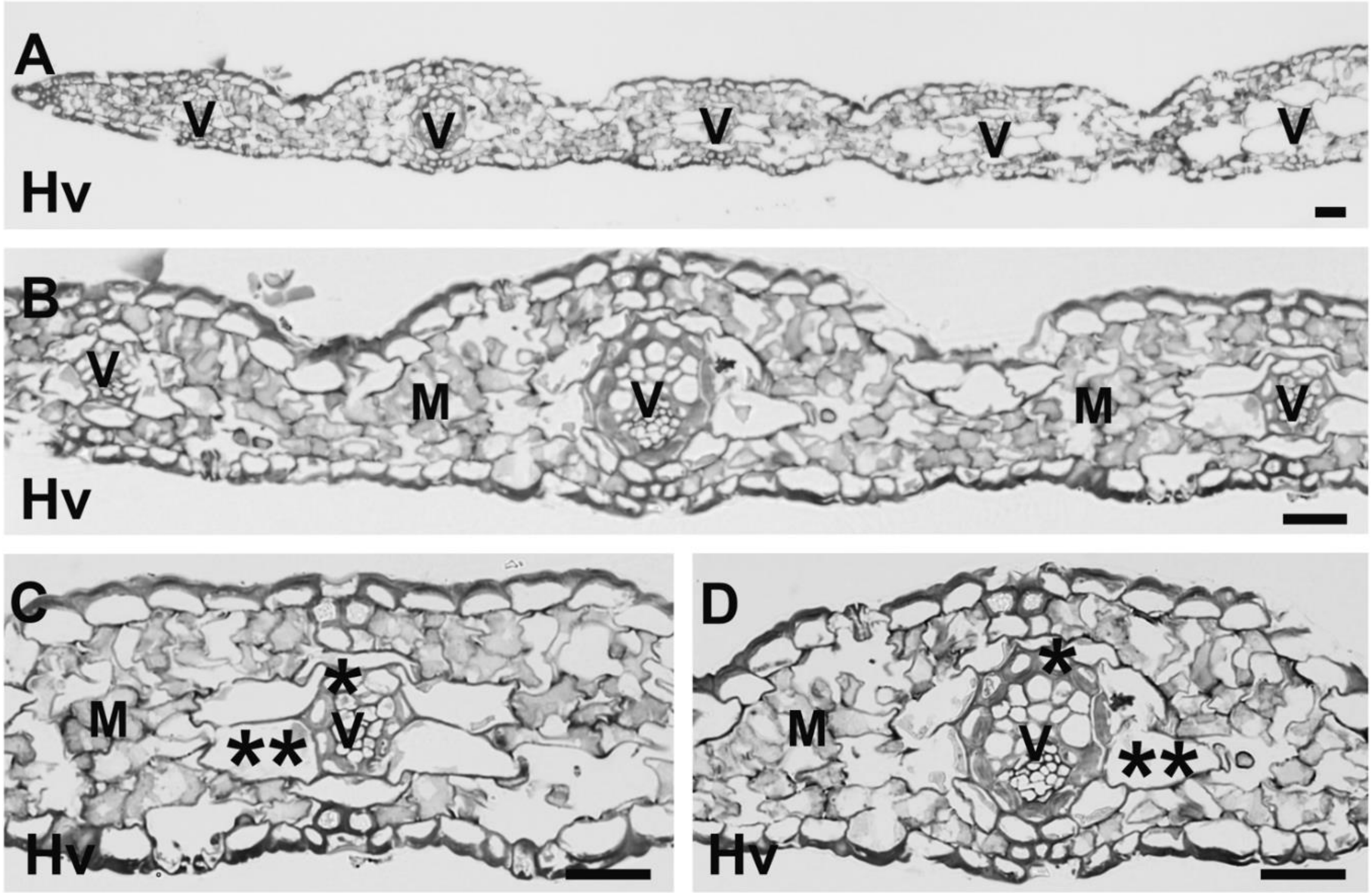

Supplementary Figure S1. Internal leaf anatomy of *Homolepis villaricensis* (Hv, C_3). Scale bar. 20 μm. M, mesophyll; V, vascular tissue. Single asterisk, inner sheath; double asterisks, outer sheath.

Pereira Alvarenga *et al*. Supplementary Figure S2

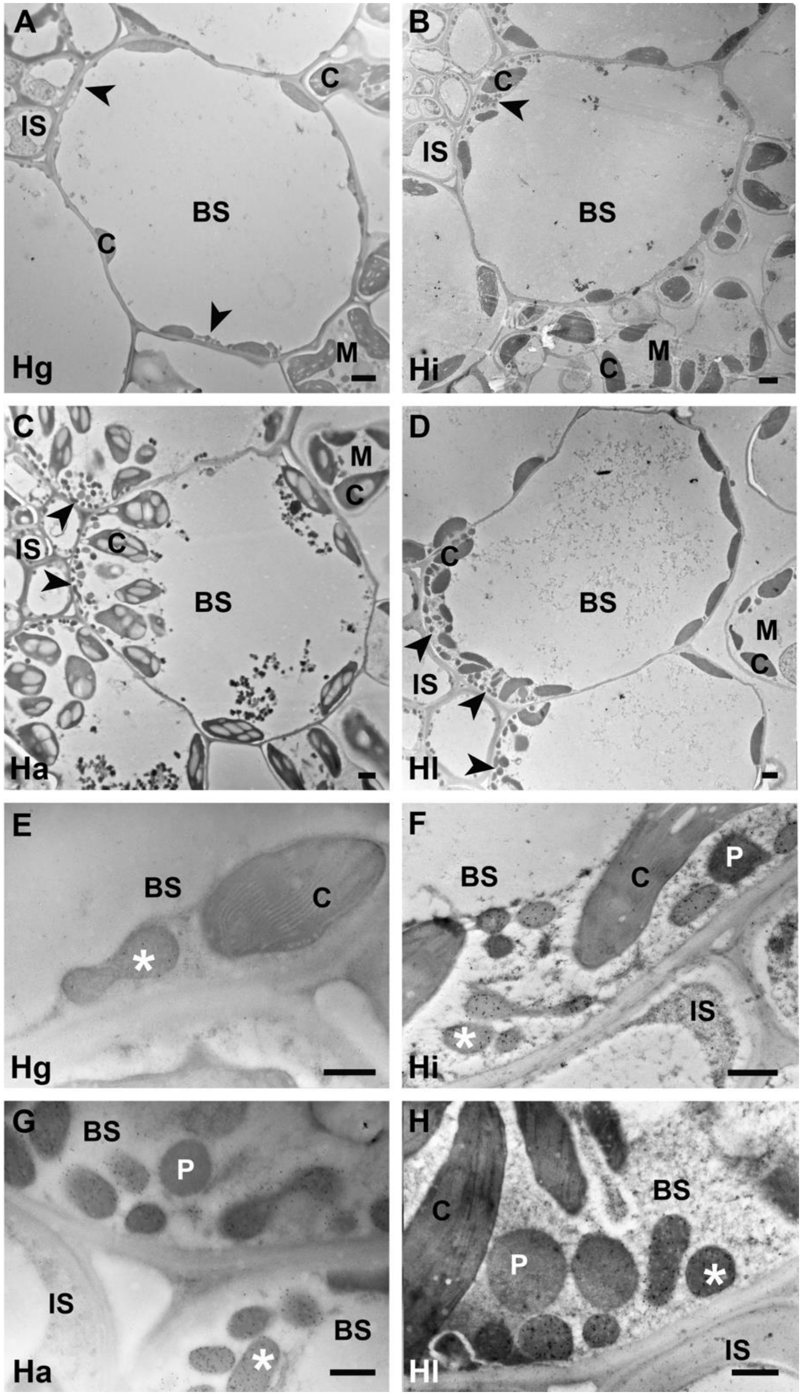

Supplementary Figure S2. Transmission electron micrographs of the bundle sheath of *Homolepis* species (A-D) and immunogold labeling of GLDP (black dots, E-H) in bundle sheath cells of *Homolepis* species. Scale bar. A-D, 2 µm; E-H, 0.5 µm. BS, bundle sheath; C, chloroplast; IS, inner sheath; M, mesophyll; P, peroxisome; Asterisk, mitochondrion. Black arrowhead (A-D) highlights mitochondria and peroxisomes. Hg, *H. glutinosa* (C_3); Hi, *H. isocalycia* (sub-C_2); Ha, *H. aturensis* (C_2); Hl, *H. longispicula* (C_2).

Pereira Alvarenga *et al*. Supplementary Figure S3

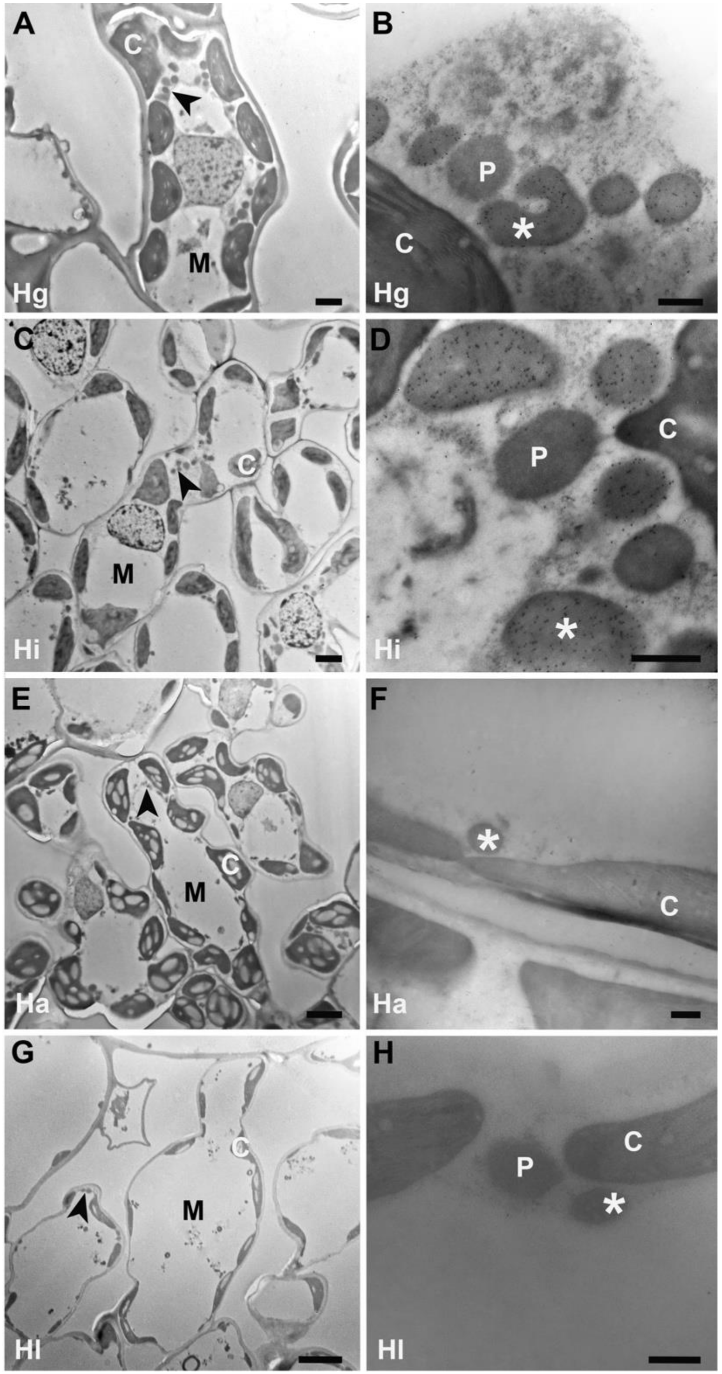

Supplementary Figure S3. Transmission electron micrographs of *Homolepis* species mesophyll and immunogold labeling of GLDP (black dots, B, D, F, H) in *Homolepis* species mesophyll cells. Scale bar. A, C, E, G, 2 μm; B, D, F, H, 0.5 μm; C, chloroplast; M, mesophyll; P, peroxisome; Asterisk, mitochondrion. Black arrowhead highlights mitochondria and peroxisomes. Hg, *H*. *glutinosa* (C_3); Hi, *H*. *isocalycia* (sub-C_2); Ha, *H*. *aturensis* (C_2); Hl, *H*. *longispicula* (C_2).

Pereira Alvarenga *et al*. Supplementary Figure S4

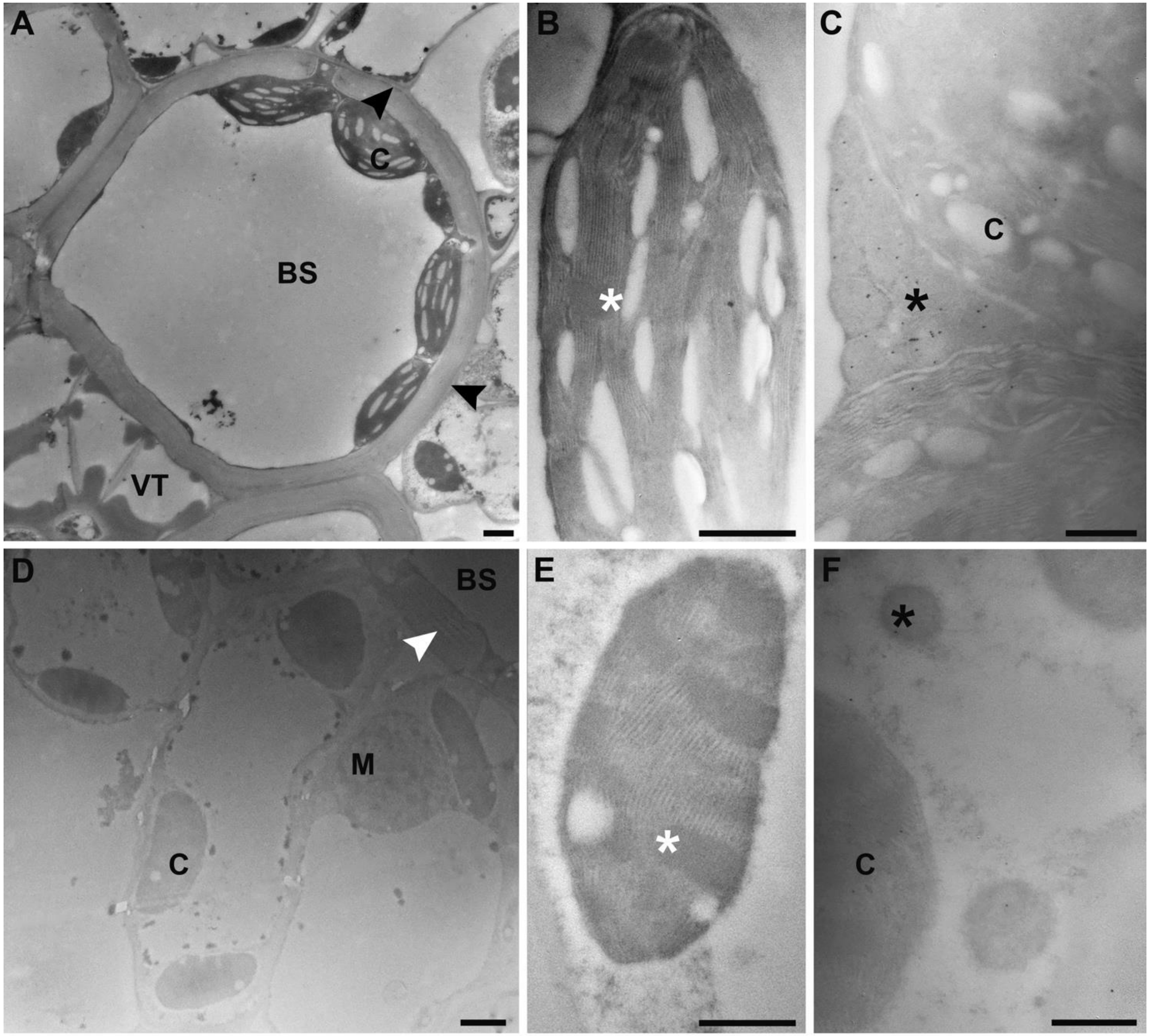

Supplementary Figure S4. Transmission electron micrographs of *Mesosetum loliiforme* (C_4) bundle sheath (A-C) and mesophyll (D-F) cells, a single chloroplast from each cell type (B, E) and immunogold labeling of GLDP (black dots) in both cell types (C, F). Scale bar. A-C, 2 µm; D-F, 0.5 µm. BS, bundle sheath; C, chloroplast, M, mesophyll; VT, vascular tissue. Black asterisk, mitochondrion. White asterisk, granum. White arrowhead, lamellate wall of bundle sheath; Black arrowhead, suberin layer.

Supplementary Figure S5. Transmission electron micrographs illustrating the absence (*Homolepis* species A-H) and presence (*Mesosetum* species I-K) of a suberin layer in the bundle sheath cell wall. A, C, E, G, I, 2 µm; B, D, F, H, J, K, 0.5 µm. BS, bundle sheath; M, mesophyll; *, bundle sheath cell wall; Arrowhead, suberin layer; Arrow, middle lamella between bundle sheath and mesophyll cells. Hg, *H. glutinosa* (C_3); Hi, *H. isocalycia* (sub-C_2); Ha, *H. aturensis* (C_2); Hl, *H. longispicula* (C_2); *Mesosetum loliiforme* (C_4); MF *Mesosetum ferrugineum* (C_4).

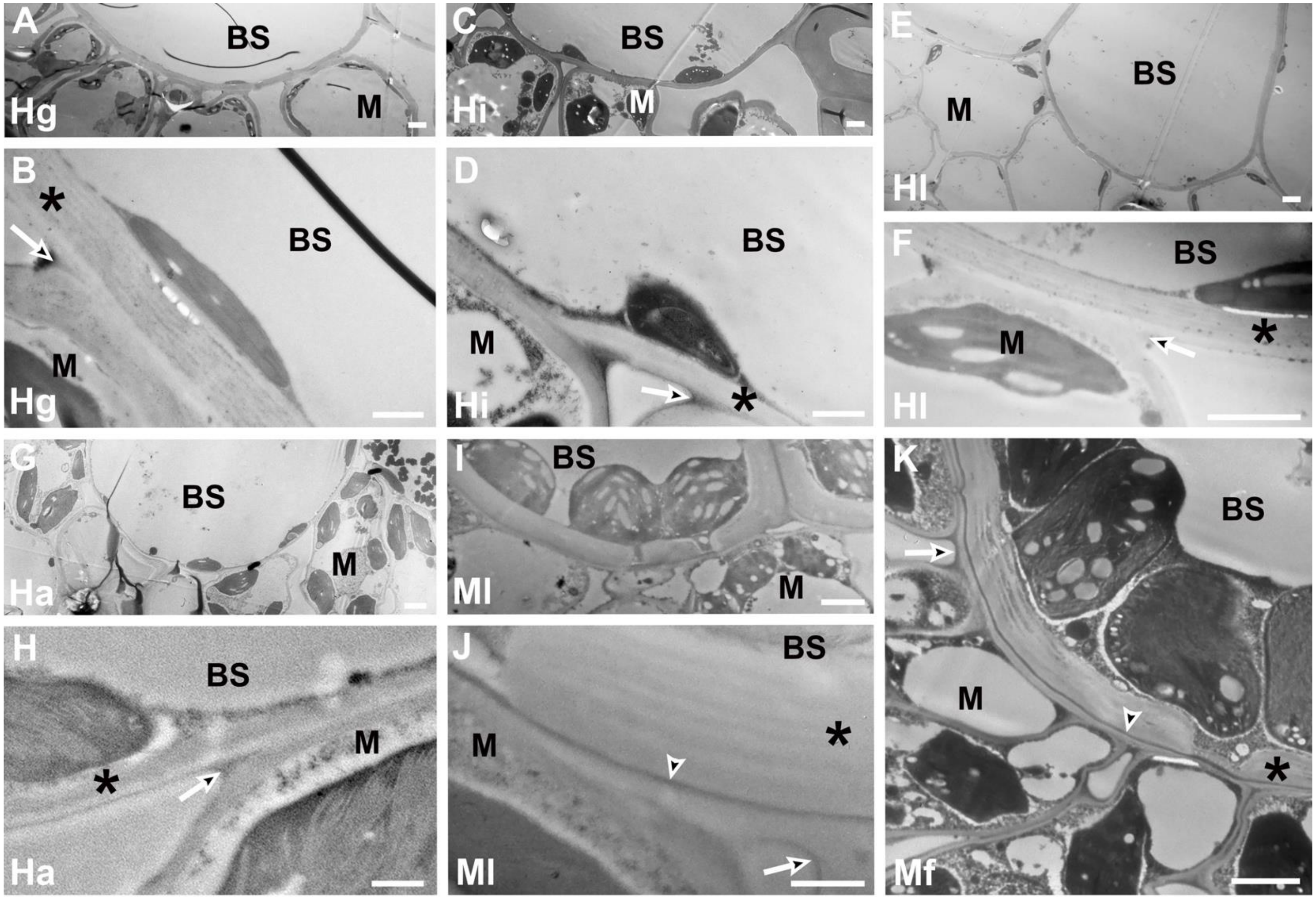

Pereira Alvarenga *et al*. Supplementary Figure S6

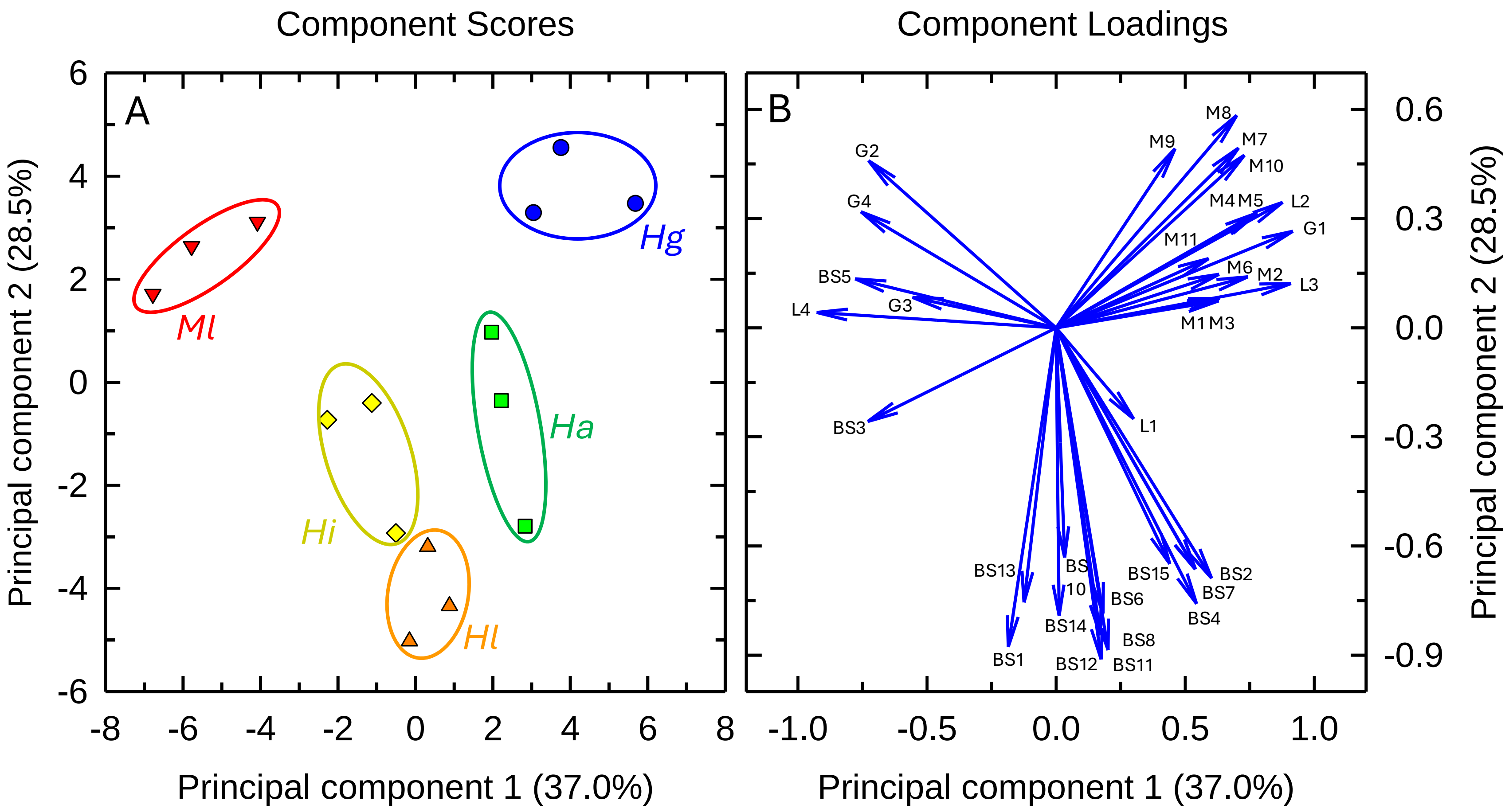

Supplementary Figure S6: Plots of a principal component analysis for parameters of the species measured in this study, where panel A shows component scores for *Mesosetum loliiforme* (*Ml*), *Homolepis aturensis* (*Ha*), *H. glutinosa* (*Hg*), *H. isocalycia* (*Hi*) and *H. longispicula* (*Hl*). Panel B shows the corresponding component loadings for the data in the analysis. See next page for component abbreviations.

Pereira Alvarenga *et al*. Supplementary Figure S6 continued: Component Loading Abbreviations

Code	Component Loading	Code	Component Loading
BS1	Chloroplast number per BS cell area	G3	A_{400}/A_{sat}
BS2	% Inner chloroplast number	G4	A/g_s (intrinsic WUE)
BS3	Chloroplast area per BS cell area, %	L1	Area per BS cell
BS4	% Chloroplast area in inner BS	L2	Interveinal distance
BS5	Area per chloroplast	L3	Mesophyll to bundle sheath area ratio
BS6	Mitochondria number per BS cell area	L4	Vein density
BS7	% Inner mitochondria number	M1	Chloroplast number per M cell area
BS8	Mitochondria area per BS cell area, %	M2	Chloroplast area per M cell area, %
BS9	% Mitochondria area in inner BS	M3	M chloroplast size
BS10	Mitochondria size	M4	Mitochondria number per M cell area
BS11	Total GLDP dots per BS cell area	M5	Mitochondria area per M cell area, %
BS12	GLDP dots per mitochondria area	M6	Area per mitochondria
BS13	Number of peroxisome per BS cell area	M7	Total GLDP dots per M cell area
BS14	% peroxisome area per BS cell area	M8	GLDP dots per M mitochondria area
BS15	Area per peroxisome	M9	Peroxisome number per M cell area
G1	CO_2 compensation point, Γ	M10	Peroxisome area per M cell area, %
G2	Initial slope of the A/C_i curve	M11	peroxisome size

See Tables 1-4 for quantities and units. Area refers to planar area in section. Parameter codes refer to bundle sheath data (BS); gas exchange data (G); leaf structure data (L), mesophyll data (M). Abbreviations: *A*, net CO_2 assimilation rate, C_i, intercellular CO_2 concentration; GLDP, glycine decarboxylase P subunit; g_s, stomatal conductance.

Pereira Alvarenga *et al*. Supplementary Figure S7

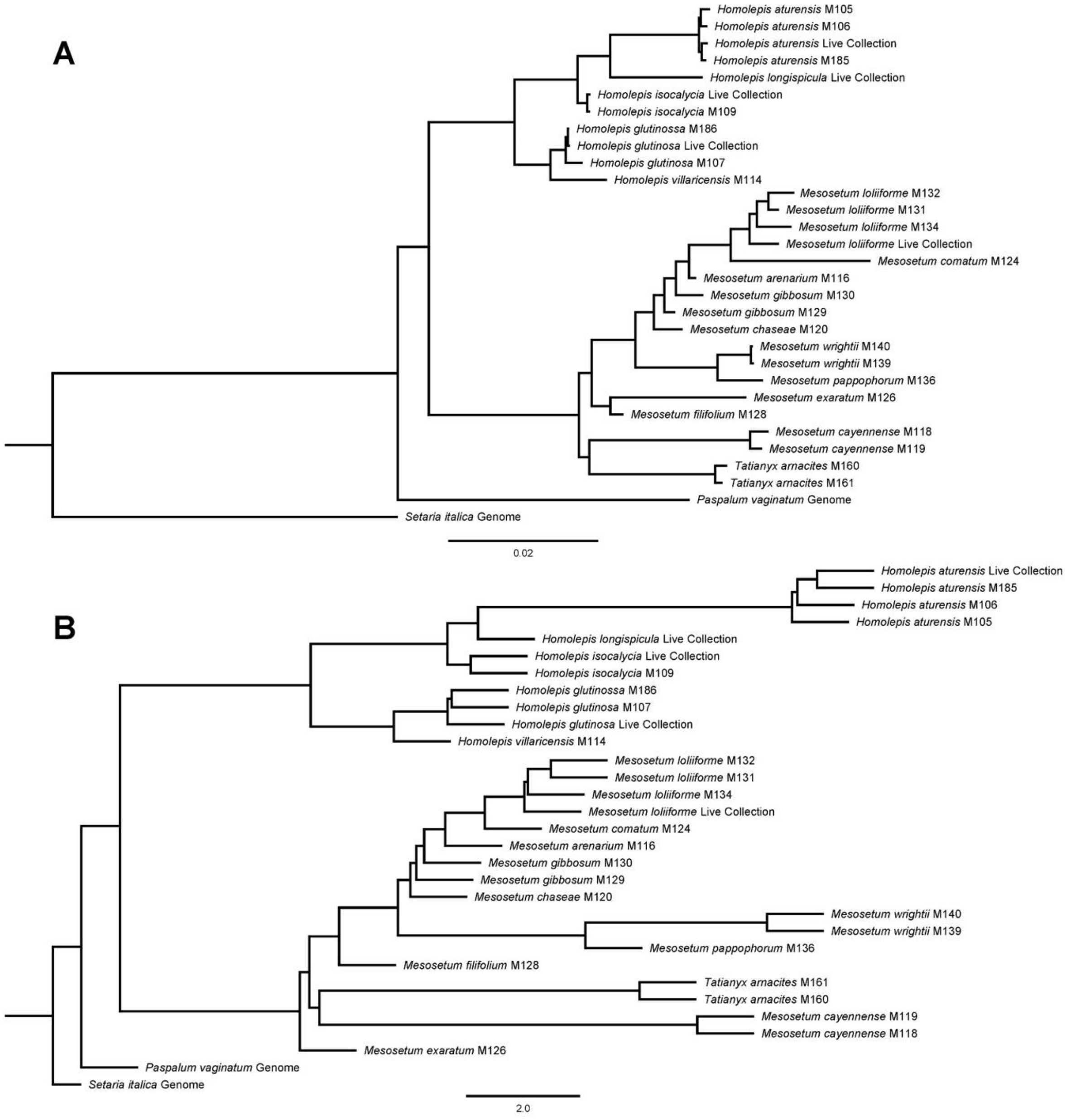

Supplementary Figure S7: Phylogenetic trees for *Homolepis* and *Mesosetum* generated with a maximum likelihood phylogenetic inference based on a concatenated super-matrix of 2,858 genes (A) and a coalescent-based phylogenetic inference based on 4,189 gene trees (B). The trees are summarized in Fig. 8 of the main text. All nodes in both trees have 100% bootstrap support (A) or 1.0 local posterior probability (B). Branch lengths and scale bars indicate substitution rates (A) and coalescent units (B). Note that all tip branches in B are set to 1 coalescent unit by default. At the species level, both trees exhibit the same topology for the *Homolepis* clade. Some incongruence exists between the two trees within the *Mesosetum* clade, which is not the main focus of this study.
